# Operando-Defined Amorphous Energy Materials for Electrochemical Energy Systems: From Structural Definition to Descriptor-Guided Design

**DOI:** 10.3390/ma19153280

**Published:** 2026-08-03

**Authors:** Lijun Chen, Wenxiu Li, Xueli Zhang, Yantao Zhao

**Affiliations:** Shanxi Research Institute of Huairou Laboratory, Taiyuan 030032, China; chenlijun@sxri.hrl.ac.cn (L.C.); liwenxiu@sxri.hrl.ac.cn (W.L.); zhangxueli@sxri.hrl.ac.cn (X.Z.)

**Keywords:** amorphous energy materials, working-state structure, operando characterization, dynamic reconstruction, solid-state ionic devices

## Abstract

Amorphous materials offer distinctive opportunities for electrochemical energy conversion and storage because their local coordination, structural flexibility and metastability can promote catalysis, ion transport and charge storage. However, amorphousness is often inferred only from broad diffraction features or missing lattice fringes, which cannot distinguish genuine amorphous phases from poorly crystalline, defect-rich, surface-amorphized or reconstructed structures. This review establishes an operando-defined framework for understanding and designing amorphous energy materials. We first clarify their structural hierarchy and identify short-range order, medium-range connectivity, interfacial heterogeneity and reconstruction propensity as key descriptors. We then examine how synthesis controls disorder and how multimodal characterization, operando measurements and experiment-constrained modeling can resolve local and working-state structures. Representative applications in electrocatalysis, batteries, supercapacitors, solid electrolytes and solid oxide electrochemical devices are discussed through structure–performance relationships. Finally, we propose descriptor-guided interface engineering and physics-informed data-driven strategies for rational design. This framework moves the field beyond the empirical “XRD-amorphous” label toward quantitative control of dynamically evolving functional structures.

## 1. Introduction

Electrochemical energy technologies require materials that remain active and stable under coupled chemical, electrochemical and mechanical stresses. Materials design has traditionally focused on crystalline solids because their periodic structures enable clear structure–property relationships. Yet many high-performance energy materials deviate from ideal crystallinity during operation, forming defect-rich domains, amorphous surface layers, reconstructed active phases or amorphous/crystalline interfaces. This has shifted amorphous energy materials from being viewed as poorly crystallized by-products to a distinct class of functional solids [1,2,3,4].

Amorphous materials lack long-range periodic order, but they are not structurally featureless. They often retain short-range coordination motifs, medium-range connectivity, compositional heterogeneity and metastable local units. In energy applications, these local structures are frequently more important than crystallographic symmetry because catalytic reactions, ion transport and redox processes occur in local coordination environments. As a result, amorphous structures can increase unsaturated sites, broaden ion pathways, accommodate strain and adaptively reconstruct under bias or cycling [5,6,7].

Amorphous materials are widely used in water splitting, oxygen electrocatalysis, CO_2_ reduction, batteries and supercapacitors [8,9,10,11,12,13]. However, their reported advantages arise from different structural origins, including intrinsic short-range order, surface amorphization, electrolyte-induced reconstruction and cycling-induced interphase formation [14,15,16,17,18]. Grouping these phenomena under a generic “amorphous enhancement” concept obscures the real mechanisms.

Amorphous energy materials also pose three persistent challenges. First, weak XRD peaks, broad diffraction humps or missing TEM lattice fringes cannot reliably distinguish amorphous phases from nanocrystalline, turbostratic, defect-rich or surface-amorphized materials. Second, amorphous structures are heterogeneous and often evolve during operation; the real active phase may be an operando-generated oxyhydroxide, interphase or reconstructed shell rather than the as-prepared material. Third, the lack of long-range order makes it difficult to define quantitative descriptors comparable to lattice parameters, facets or point defects [19,20].

**Figure 1:**
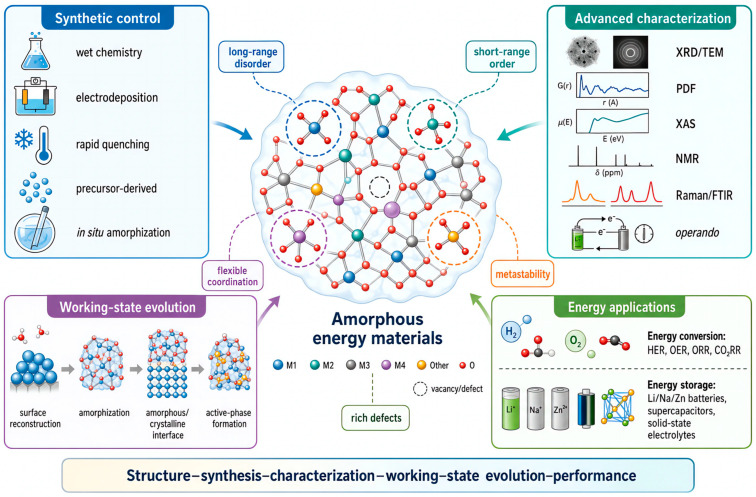
Conceptual framework of amorphous energy materials. Amorphous energy materials demand holistic analysis across five interwoven dimensions: structural disorder, synthetic regulation, multimodal characterization, working-state structural evolution, and practical energy applications. During the preparation of this figure, the authors used OpenAI ChatGPT (version 5.5) to assist in generating the initial visual layout and graphical elements. The authors reviewed, revised, and scientifically validated all graphical contents and take full responsibility for the final figure.

This review therefore asks a stricter question: how can amorphous energy materials be defined, monitored and rationally designed under working conditions? The conceptual shift must move from a diffraction-based amorphous label to an operando-defined, descriptor-based and designable working-state structure. Figure 1 then operationalizes this idea by linking synthesis-controlled disorder, structural descriptors, multimodal characterization, working-state evolution and electrochemical function. This framework shifts the review from an application catalogue to an operando-defined and descriptor-guided view of amorphous energy materials.

## 2. Structural Nature of Amorphous Energy Materials: From Phase Label to Working-State Descriptor

**Figure 2:**
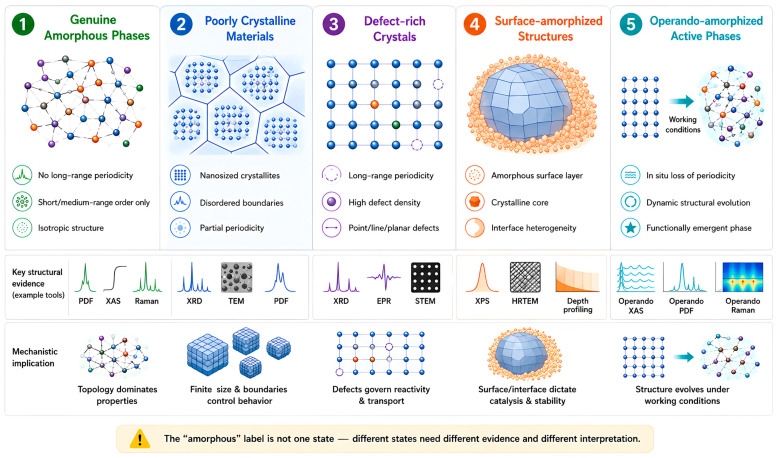
Structural states behind the amorphous label. Genuine amorphous phases, poorly crystalline materials, defect-rich crystals, surface-amorphized structures and operando-amorphized active phases require different structural evidence and mechanistic interpretation. During the preparation of this figure, the authors used OpenAI ChatGPT (version 5.5) to assist in generating the initial visual layout and graphical elements. The authors reviewed, revised, and scientifically validated all graphical contents and take full responsibility for the final figure.

A central problem in amorphous energy material research is the overuse of the term “amorphous”. As summarized in Table 1, weak XRD peaks, broad diffraction features or missing TEM lattice fringes are useful screening signals but cannot by themselves distinguish genuine amorphous solids from poorly crystalline nanoparticles, defect-rich crystals, surface-amorphized core–shell structures or phases that amorphize only during operation (Figure 2). This distinction is mechanistically important. In electrocatalysis, the active phase is often an in situ-generated oxyhydroxide rather than the pristine precursor; in batteries, cycling can induce amorphization while retaining redox-active local motifs; in solid electrolytes, disordered networks follow transport mechanisms different from crystalline vacancy channels. A complete structural description must therefore include long-range order, local bonding, nanoscale heterogeneity and working-state evolution.

### 2.1. Long-Range Disorder and Short-Range Order

Amorphous solids are defined by the absence of long-range translational periodicity, typically reflected by broad XRD halos rather than sharp Bragg peaks. They nevertheless retain chemically meaningful local order. Since Zachariasen’s random-network model and Elliott’s concept of medium-range order, it has become clear that noncrystalline materials cannot be described as random atomic aggregates [21,22]. Total scattering, PDF analysis, XAS and atomistic modeling further confirm that amorphous solids preserve short-range coordination units and partially ordered medium-range connectivity [23,24,25,26].

The coexistence of long-range disorder and short-range order is the functional core of amorphous energy materials. Electrocatalysis, ion insertion, charge compensation and interfacial reconstruction are governed by local coordination rather than periodic symmetry. Amorphous transition-metal oxides and oxyhydroxides contain distorted MO_x_ polyhedra, variable metal valence, undercoordinated sites and interconnected metal–oxygen networks that tune adsorption, redox flexibility and proton-coupled electron transfer [27]. Similarly, amorphous MoS_x_ retains local Mo-S coordination and terminal/bridging sulfur motifs correlated with HER activity [28,29].

The same principle applies to energy storage electrodes. Amorphous TiO_2_, V_2_O_5_, FePO_4_ and related insertion hosts retain local redox-active motifs despite losing long-range order. Li^+^, Na^+^, Zn^2+^ and multivalent-ion transport is controlled by metal–oxygen polyhedra, free volume, bond-length distributions and pathway connectivity [30,31]. Surface-amorphized TiO_2_ illustrates this balance: the disordered shell accelerates Li^+^ access, while the crystalline core preserves structural integrity [30]. Amorphous vanadium oxide similarly improves sodium storage through a more open and accessible framework [32].

Three structural dimensions are therefore essential: short-range order, including coordination number, bond length, bond-angle distribution and oxidation state; medium-range connectivity, describing how local polyhedra or clusters are linked; and nanoscale heterogeneity, including composition gradients, amorphous/crystalline interfaces, hydration, residual ligands, pores and reconstructed surfaces. These descriptors are more useful than the vague label of “XRD-amorphous”.

### 2.2. Classification of Amorphous, Disordered, Poorly Crystalline and Operando-Amorphized Phases

The existing literature frequently confuses amorphous, disordered, poorly crystalline, defect-rich and reconstructed phases, which correspond to distinct structural states and lead to divergent mechanistic interpretations, necessitating strict classification.

Genuine amorphous materials feature complete local chemical order and no long-range translational order, characterized by broad XRD halos, diffuse SAED rings and absent continuous HRTEM lattice fringes. Reliable structural identification relies on local characterization techniques, including PDF, EXAFS, Raman and solid-state NMR. For instance, amorphous Co-based phosphate/oxide OER catalysts are not unstructured deposits; spectroscopic tests confirm their dynamic local structural and oxidation state evolution under catalytic conditions [7].

Poorly crystalline materials consist of nanocrystalline domains with limited lattice coherence. Their broad XRD Bragg peaks originate from tiny crystallite size, lattice strain or stacking disorder, easily leading to conventional XRD misidentification as amorphous. Residual lattice periodicity can be detected via HRTEM, long-distance PDF signals and synchrotron total scattering. The catalytic activity of poorly crystalline oxides and hydroxides stems from exposed crystal facets, grain boundaries and nanodomains, rather than genuine amorphous networks.

Defect-rich crystals maintain intact crystallographic frameworks with inherent vacancies, dopants, dislocations, stacking faults, antisite defects and lattice strain. Their diffraction patterns retain distinguishable Bragg peaks with slight broadening, representing local disorder superimposed on ordered lattices. Defect engineering is widely applied in energy materials, but the enhanced activity of defective crystals cannot be attributed to amorphization. For example, oxygen vacancies in crystalline metal oxides modulate electronic structure and adsorption energy while preserving overall lattice order [33,34]; sulfur vacancies and exposed edges boost the HER activity of defective crystalline MoS_2_, which is structurally distinct from amorphous MoS_x_ [29,35].

Surface-amorphized materials possess a crystalline core coated with an amorphous shell, a ubiquitous structure in nanomaterials and battery electrodes. The crystalline core dominates bulk XRD signals, while surface-sensitive TEM, XPS and EXAFS effectively detect the amorphous outer layer. This core–shell structure synergizes the amorphous shell’s fast ion transport and optimized redox kinetics with the crystalline core’s electronic conductivity and mechanical stability, with surface-amorphized TiO_2_ serving as a classic high-rate lithium storage prototype [30].

Operando-amorphized materials are substances that are initially crystalline or semi-crystalline and transform into amorphous or highly disordered active phases during electrochemical operation, which is prominent in electrocatalysis. Most transition-metal phosphides, sulfides, nitrides and borides are not intrinsic OER active phases; anodic potentials drive their surface reconstruction into amorphous/poorly crystalline metal oxyhydroxide layers [36,37]. Operando XAS and Raman techniques verify that these reconstructed layers dominate catalytic activity. In NiFe oxyhydroxide systems, operando XAS identifies distorted octahedral Fe sites as high-efficiency OER centers, demonstrating that working-state structure outweighs initial crystalline/amorphous attributes [38,39]. This paradigm shifts modern electrocatalysis research toward operando-defined active phases [37,40].

In short, broad diffraction signals are not exclusive markers of amorphousness, as they can also derive from ultra-small nanocrystals, stacking disorder, electron-beam-sensitive domains, surface amorphization and electrochemical reconstruction. Accurate structural classification requires the integration of diffraction, microscopy, local structure analysis and operando characterization. The core conceptual differences between genuine amorphous and disordered/crystalline derived materials are summarized in Table 2.

### 2.3. Structural Descriptors for Amorphous Energy Materials

Without conventional crystallographic descriptors such as lattice parameters and Miller-indexed facets, amorphous materials require statistical structural parameters to quantify local features, including coordination number, bond-length/bond-angle distribution, pair correlation peak characteristics, local oxidation state, density of states, free volume, medium-range connectivity, chemical heterogeneity and structural relaxation degree [41].

For electrocatalysts, local coordination and oxidation state are the most critical descriptors. In OER catalysts, M–O bond length, metal valence, M–O–M connection mode, oxygen vacancy disorder and high-valence oxyhydroxide formation capability determine catalytic performance. The in situ-formed CoPi catalyst exists as a disordered cobalt oxide/phosphate network under near-neutral conditions [42], with catalytic behavior controlled by proton-coupled electron transfer, cobalt valence variation and electrolyte-derived phosphate groups [43,44]. For NiFe oxyhydroxides, Fe doping markedly enhances OER activity; operando XAS confirms that Fe occupies shortened octahedral Fe–O sites in the NiOOH matrix, verifying that local coordination rather than crystallinity dictates activity [45].

HER catalyst performance is governed by metal–sulfur coordination, terminal sulfide/disulfide content, unsaturated metal centers and electronic conductivity. Amorphous MoS_x_ exhibits diverse Mo–S local environments absent in crystalline MoS_2_. In situ XAS confirms that terminal disulfide species participate in proton reduction, correlating local sulfur motifs with HER activity [46]. Unlike crystalline catalysts relying on single ideal active sites, amorphous catalysts require statistical description of diverse local structural distributions.

Battery electrode descriptors focus on free volume, ion coordination environment, diffusion pathway percolation, redox center distribution, structural water and framework flexibility. Amorphous electrodes enable isotropic ion transport but suffer from low electronic conductivity and irreversible side reactions; thus, they are usually coupled with conductive skeletons, partial crystalline domains and stable interfacial layers to balance ion accessibility and structural durability [47]. For solid electrolytes, key descriptors include network composition, pore bottleneck size, polarizability, ion site distribution and interfacial stability. Amorphous LiPON retains practical value in thin-film batteries via superior lithium metal compatibility despite moderate ionic conductivity [48], while sulfide glass electrolytes form continuous ion transport pathways and achieve excellent electrode contact via disordered networks [49].

No single characterization technique can capture all structural descriptors. XRD and electron diffraction probe long-range order but not local bonding details; PDF analysis characterizes short-/medium-range atomic correlations; XAS provides element-specific oxidation state and coordination information for amorphous catalysts and electrodes; solid-state NMR identifies local elemental environments in amorphous electrolytes; Raman and FTIR capture local bond vibration and surface species signals. Reliable structural assignment depends entirely on multimodal collaborative characterization.

Overall, amorphous energy materials form a continuous structural spectrum rather than a crystal–amorphous binary division. Fully crystalline materials with complete periodicity and pure amorphous networks with only local order occupy two ends, with poorly crystalline, defect-rich, surface-amorphized and operando-reconstructed structures distributed in between. This structural hierarchy explains material performance differences, the poor reproducibility of amorphous systems and the necessity of operando characterization for real working-structure identification.

## 3. Mechanisms for Amorphous Structures to Boost Electrochemical Performance

The electrochemical advantages of amorphous energy materials arise from coupled structural and electronic effects rather than disorder alone. Long-range disorder, retained short-range coordination, distorted bonding, metastability and compositional heterogeneity diversify active sites, broaden adsorption energy distributions, enable flexible redox centers, reduce directional diffusion constraints and improve strain tolerance [23,34,41]. These coupled effects are especially important for proton–electron–ion coupled processes.

### 3.1. Abundant Active Sites and Flexible Coordination Environments

Long-range disorder creates undercoordinated atoms, dangling bonds, distorted polyhedra, local vacancies and high-energy domains. More importantly, it broadens coordination and adsorption energy distributions, which benefits multi-step reactions such as OER, HER, ORR and CO_2_RR that require different binding strengths for different intermediates.

Amorphous Co/Ni/Fe-based oxyhydroxides are typical high-performance OER catalysts. The anodically formed CoPi catalyst delivers excellent near-neutral water oxidation activity, with performance jointly determined by cobalt–oxygen networks, variable valence, proton-coupled electron transfer and phosphate buffering [36,42,44]. NiFe oxyhydroxide systems further validate this mechanism: trace Fe doping decisively enhances OER activity, with operando XAS confirming unique coordinated Fe sites as the core active centers. Amorphous networks stably host high-valence metal centers and distorted M–O structures that are inaccessible in rigid crystalline lattices.

For HER, amorphous MoS_x_ is the most representative non-noble catalytic system. Electrodeposited amorphous MoS_x_ films exhibit high-efficiency pH-universal HER activity, with sulfur-rich local motifs and electrochemical activation dominating performance [50]. In situ XAS verifies terminal disulfide-mediated proton reduction, highlighting the key role of diverse unsaturated Mo–S active environments in amorphous systems, which outperform crystalline MoS_2_ with edge-site-limited activity [50]. Flexible coordination also enables amorphous phosphides, borides and metal–metalloid alloys to serve as ideal pre-catalysts for overall water splitting, with reversible structural reorganization adapting to HER and OER working conditions [36,37].

### 3.2. Tunable Electronic Structure and Metastability

Most amorphous phases are metastable and can rearrange under electrochemical bias. Unlike crystalline materials constrained by periodicity, amorphous solids contain distributed bond lengths, local compositions and redox environments, producing broader electronic states and more continuous redox-potential distributions [51]. This can optimize intermediate binding and accelerate interfacial charge transfer.

Catalytic activity follows adsorption energy scaling relationships, with optimal performance requiring moderate intermediate binding strength [52,53]. Though unable to break intrinsic scaling rules, amorphous structures expand local adsorption environments and increase the proportion of optimally active sites, which is critical for OER involving dynamic ·OH, ·O and ·OOH intermediate conversion and metal valence-bond coupling [54].

Amorphous metal oxides and oxyhydroxides with variable valence and structural distortion facilitate catalytic redox transitions. Anodic potentials induce high-valence metal–oxygen active species in Co-based and NiFe-based OER catalysts, and flexible amorphous networks reduce the energy barrier for bond contraction, valence variation and proton removal compared with rigid crystalline frameworks [44,54]. Similarly, amorphous MoS_x_ acts as an adaptive dynamic system: electrochemical activation continuously optimizes sulfur species and Mo coordination structures, stabilizing high-activity configurations [50].

Notably, metastability is a double-edged sword. Excessive disorder causes material dissolution, irreversible phase transition, conductivity loss and structural collapse. Thus, optimal amorphous modification requires balanced metastability and structural/electronic/mechanical stability, with high-performance materials featuring controllable disorder rather than maximum disorder degree.

### 3.3. Fast Ion Transport and Isotropic Diffusion Pathways

In energy storage, amorphous structures promote ion transport and strain tolerance. Crystalline electrodes rely on anisotropic and size-limited channels, whereas amorphous frameworks provide more isotropic pathways, abundant insertion sites and expanded free volume. These features are particularly useful for large-radius or multivalent ions such as Na^+^, K^+^, Zn^2+^, Mg^2+^ and Al^3+^.

Surface-amorphized TiO_2_ exemplifies the advantages of amorphous/crystalline composite structures: the disordered surface accelerates Li^+^ interfacial transport, while the crystalline core maintains structural integrity, achieving superior high-rate lithium storage [47]. Amorphous vanadium oxide delivers excellent sodium storage via open flexible disordered frameworks with abundant accessible insertion sites [55]. In aqueous Zn-ion batteries, controlled partial amorphization of vanadium oxide cathodes reduces lattice electrostatic constraints, improving Zn^2+^ diffusion kinetics during cycling [56].

Amorphous manganese oxides are widely applied in Zn-ion batteries and supercapacitors, with structural disorder and hydration promoting multivalent ion mobility and rapid surface redox reactions [24]. For pseudocapacitive energy storage, amorphous/poorly crystalline oxides eliminate slow solid-state diffusion, realizing high-rate charge storage via unobstructed ion transport and diverse redox-active sites. However, excessive disorder reduces electronic conductivity, induces irreversible capacity loss and accelerates electrolyte decomposition. Optimized amorphous storage materials integrate conductive skeletons, partial crystalline domains and stable coatings to balance ion accessibility and interfacial stability.

### 3.4. Strain Accommodation and Structural Buffering

Repeated charge–discharge cycling causes severe volume expansion/contraction, particle pulverization and unstable SEI films in conversion-/alloying-type battery electrodes. Amorphous structures alleviate mechanical damage via local bond rotation, reversible bond breaking/reformation and short-range structural rearrangement, avoiding long-range lattice fracture and serving as a critical guarantee of cycling stability.

Transition-metal oxide anodes evolve into disordered metal/Li_2_O composite phases after cycling, with structural disorder buffering volume fluctuation and supporting reversible conversion-type redox reactions [47]. Silicon-based anodes further validate the strain regulation advantage of amorphization: amorphous silicon homogenizes lithiation/delithiation stress and eliminates anisotropic volume expansion of crystalline silicon, suppressing electrode fracture [57].

Amorphous/glassy solid electrolytes also possess superior structural compliance. Amorphous LiPON thin films form stable electrode–electrolyte interfaces and exhibit excellent lithium metal compatibility for thin-film batteries [58]. Sulfide glass electrolytes such as Li_2_S–P_2_S5 achieve high ionic conductivity and better electrode mechanical contact than brittle crystalline oxide ceramics [59]. Despite the ultrahigh conductivity of crystalline LGPS electrolytes, amorphous electrolytes remain competitive due to outstanding processability, deformability and interfacial compatibility [60].

Nevertheless, structural buffering is not unlimited. Excessively flexible amorphous frameworks undergo continuous relaxation, dissolution and side reactions, leading to voltage hysteresis and capacity attenuation. The optimal amorphous structure balances local strain-accommodating flexibility and stable chemical connectivity for reversible redox pathways.

### 3.5. Dynamic Reconstruction as a Source of Catalytic Activity

A core modern consensus is that most amorphous materials act as pre-catalysts rather than final active phases, achieving high activity via operando dynamic structural reconstruction. In OER electrocatalysis, crystalline transition-metal phosphides, sulfides, selenides, nitrides and borides undergo surface oxidation under anodic potentials, reconstructing into amorphous/poorly crystalline metal oxyhydroxide active layers [37]. The pristine precursor provides a conductive skeleton, while the reconstructed amorphous layer dominates catalytic performance.

This mechanism reinterprets high-efficiency OER catalyst behavior. For example, Ni_2_P nanoparticles’ OER activity originates mainly from in situ-generated oxyhydroxide surface layers rather than the pristine phosphide phase. Similar reconstruction occurs in diverse metal chalcogenides and phosphides, prompting the key question of whether these pristine materials are genuine catalysts or merely active-phase precursors.

Dynamic reconstruction enhances catalysis via multiple pathways: exposing novel metal–oxygen active sites, generating high-valence metal centers, introducing defective proton/electron transfer channels, and constructing amorphous/crystalline heterointerfaces to boost charge transfer. Operando characterization is indispensable for identifying working-state evolution: operando XAS tracks metal valence and coordination changes, while operando Raman captures oxyhydroxide generation and intermediate evolution. Numerous in situ tests confirm that catalytic activity correlates more strongly with reconstructed structures than pristine materials [51]. Future research should focus on operando amorphous structure formation, structural characteristics and stability, rather than superficial pristine-phase amorphous/crystalline attributes.

Taken together, amorphous structures enhance electrochemical performance through five coupled mechanisms: diverse active sites, tunable electronic/metastable states, isotropic ion transport, strain buffering and operando reconstruction. The key challenge is to move from qualitative “disorder enhancement” to quantitative optimization of coordination distribution, bond-angle variance, valence flexibility, free volume and working-state stability. The five core performance-boosting mechanisms of amorphous energy materials are systematically summarized in Table 3.

## 4. Synthetic Strategies for Amorphous Energy Materials

**Figure 3:**
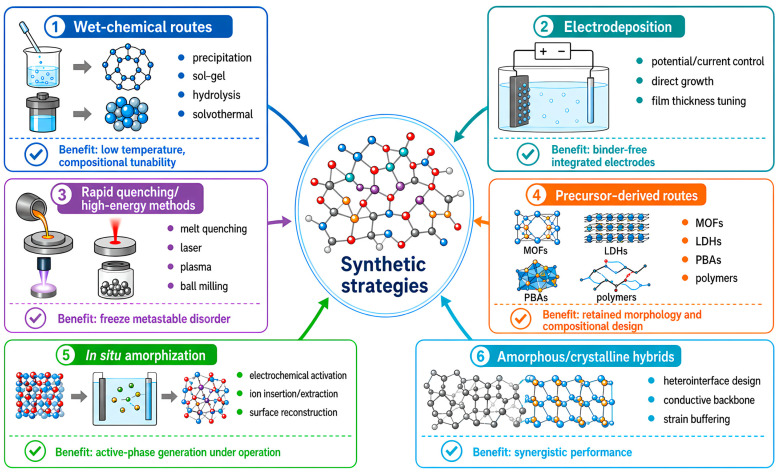
Synthetic strategies for amorphous energy materials. During the preparation of this figure, the authors used OpenAI ChatGPT (version 5.5) to assist in generating the initial visual layout and graphical elements. The authors reviewed, revised, and scientifically validated all graphical contents and take full responsibility for the final figure.

Amorphous synthesis requires suppressing long-range crystallization while preserving functional local bonding, chemical uniformity and electrochemical accessibility. Unlike crystalline synthesis, which targets phase purity and facets, amorphous synthesis relies on kinetic trapping, local-coordination retention and metastability control. The main strategies (Figure 3) include wet chemistry, electrodeposition, rapid quenching/high-energy processing, precursor conversion, in situ amorphization and amorphous/crystalline hybridization [61,62,63].

Notably, materials prepared by different methods exhibit similar weak XRD signals but distinct local bonding, medium-range order and electrochemical stability. Electrodeposited CoPi contains electrochemically induced hydrated motifs, while melt-spun amorphous alloys are kinetically frozen metallic glasses with dense atomic stacking [64,65,66]. Sol–gel-derived oxides retain residual hydroxyl groups, and electrochemically reconstructed amorphous shells couple tightly with crystalline cores. Thus, synthesis strategies must be discussed in combination with structural descriptors and working-state evolution rules.

### 4.1. Wet-Chemical Synthesis

Wet-chemical synthesis is the most widely used approach for amorphous oxides, hydroxides, sulfides, phosphates and carbon composites, covering precipitation, sol–gel, hydrolysis, solvothermal treatment, ligand-assisted synthesis and low-temperature redox reactions. Mild nucleation and condensation conditions inhibit long-range crystallization and retain metastable local coordination, hydrated structures and mixed metal components [67].

**Figure 4:**
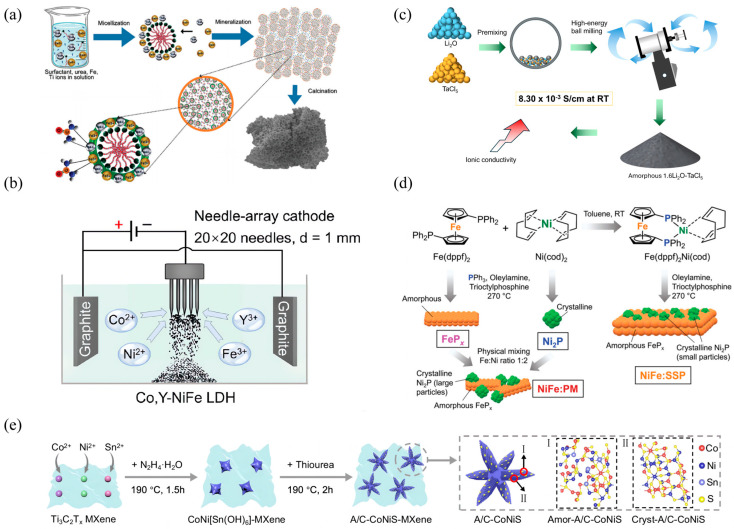
(**a**) Schematic representation of the synthesis of mesoporous honeycomb iron titanate. Adapted from Ref. [68]. (**b**) Schematic illustration of the synthesis of Co,Y-NiFeOOH by the needle-array electrodeposition method. Adapted from Ref. [73]. (**c**) Schematic illustration of the synthesis of amorphous 1.6Li_2_O-TaCl_5_ oxychloride solid electrolytes. Adapted from Ref. [77]. (**d**) Synthesis of the four different transition metal phosphide materials. Adapted from Ref. [79]. (**e**) Schematic illustration of the synthesis of A/C-CoNiS grown on Ti_3_T_2_T_x_ MXene with amorphous/crystalline interfaces. Adapted from Ref. [91].

This method produces highly dispersed metal–oxygen networks with abundant undercoordinated sites. Ashie et al. [68] fabricated amorphous mesoporous honeycomb iron titanate via sol-gel evaporation-induced self-assembly with F12 triblock copolymer as a template, and the disordered amorphous architecture delivers enlarged reactive surface and enhanced light absorption, achieving much higher photocatalytic hydrogen evolution activity than commercial TiO_2_ (Figure 4a). The local structure of amorphous Co/Ni/Fe hydroxides/oxyhydroxides is precisely tuned by pH value, counterions, aging time and post-treatment [52,53,69]. For HER, scalable wet-chemical routes fabricate nanostructured amorphous MoS_x_ with diverse Mo–S coordination, and terminal disulfide species endow the material with superior proton reduction capability [28,70].

In battery research, wet-chemical synthesis generates hydrated, porous amorphous frameworks. Solution-derived amorphous vanadium oxide delivers excellent sodium storage via unconstrained ion diffusion pathways [63], while amorphous Mn/V oxides optimize Zn^2+^ insertion kinetics in aqueous batteries via structural disorder and hydration [71,72]. The method features flexible composition regulation, low cost and scalable production, but residual ligands, adsorbed water and hydroxyl groups require strict control to avoid their interfering with electrochemical performance evaluation. Comprehensive multimodal characterization is essential for systematic structural verification.

### 4.2. Electrochemical Deposition

Unlike wet-chemical synthesis, electrodeposition inherently couples material formation with electrochemical activation. It is uniquely advantageous for binder-free amorphous electrocatalysts because conductive substrates, electrolyte composition, pH, current density and deposition mode jointly determine nucleation, film growth, oxidation state and early-stage reconstruction. This route enables adjustable film thickness, uniform loading and tight electrical contact, while also requiring careful separation of as-deposited disorder from activation-induced amorphization.

The Kanan–Nocera CoPi catalyst represents a landmark electrodeposited amorphous material, forming a dynamically repairable hydrated amorphous film under anodic polarization in neutral phosphate buffer [66]. Its catalytic function is governed by proton-coupled electron transfer, cobalt valence cycling and electrolyte-derived phosphate groups [25]. Lv et al. [73] fabricated Co/Y co-doped amorphous NiFeOOH via scalable needle-array electrodeposition, which modulates Ni/Fe hydroxyl adsorption disparities through adjusted Lewis acidity and lattice distortion to activate efficient dual-site synergistic OER pathways with superior industrial electrolysis performance (Figure 4b).

For HER, electrodeposited amorphous MoS_x_ thin films achieve stable high-efficiency catalysis across wide pH ranges, with electrolyte and deposition potential modulating local Mo–S structures and electronic properties [74]. The core advantage of this strategy is electrochemical programmability, as the growth environment closely matches practical working conditions. However, trapped electrolyte ions, uneven film thickness and substrate-induced electronic effects require systematic structural and quantitative chemical analysis.

### 4.3. Rapid Quenching and High-Energy Methods

Rapid quenching and high-energy methods fabricate amorphous materials via kinetic trapping of high-energy disordered states before atomic crystallization. Common technologies include melt spinning, splat quenching, laser processing, carbothermal shock and high-energy ball milling, widely applied in metallic glasses, metastable alloys and multi-component energy materials [75].

Bulk metallic glasses stabilize amorphous structures via multi-component composition, atomic size mismatch and negative mixing heat, integrating metallic conductivity, mechanical strength and metastable atomic arrangement for electrochemical applications [76]. Ou et al. [77] systematically studied the correlation between ball milling-induced amorphization degree and Li-ion conductivity of amorphous 1.6Li_2_O-TaCl_5_ oxychloride solid electrolytes, revealing that elevated amorphous structural defects remarkably boost room-temperature ionic conductivity up to 8.30 × 10^−3^ S/cm and offering a universal strategy to optimize amorphous electrolyte ionic transport performance (Figure 4c). Mechanical ball milling induces amorphization via severe lattice deformation and structural fracture, optimizing ion transport in battery materials, though it is limited by milling contamination and poor surface chemistry controllability [78].

These methods excel at constructing metastable disordered structures but lack precise control over porosity and nano-morphology compared with wet-chemical routes. Amorphous alloy catalysts usually require dealloying and nanostructuring to expose active sites, while mechanically prepared battery materials need conductive doping to enhance electrochemical performance.

### 4.4. Precursor-Derived Amorphous Materials

The precursor conversion strategy fabricates amorphous energy materials via thermal, chemical or electrochemical transformation of structured precursors, including MOFs, LDHs, Prussian blue analogues and polymers, enabling precise composition regulation, spatial confinement and morphology inheritance.

Mondal et al. [79] adopted a precursor-based molecular synthetic route to fabricate heterostructured NiFe phosphides built on amorphous iron phosphide matrices, and in situ spectroscopic tracking via freeze-quench technology confirmed full conversion into homogeneous NiFe oxyhydroxide after long-term OER cycling, casting doubt on the sustainability of artificial heterostructure designs for non-precious OER electrocatalysts (Figure 4d). LDH precursors with tunable metal ratios transform into amorphous/poorly crystalline oxyhydroxides during electrocatalysis, with Fe-doped NiFe LDH derivatives exhibiting superior OER activity via local disorder engineering [80]. Prussian blue analogues and polymer precursors also generate porous amorphous frameworks for ion storage and catalysis [81,82].

A key challenge of this strategy is inconsistent precursor inheritance: thermal conversion introduces residual carbon and mixed valence states, while electrochemical operation further induces structural reconstruction. Thus, research must integrate precursor structural inheritance and post-conversion dynamic evolution analysis.

### 4.5. In Situ Amorphization During Operation

Operando amorphization uses crystalline or semi-crystalline precursors to generate amorphous active layers under working conditions. This strategy is central to modern electrocatalysis. Transition-metal phosphides, sulfides, selenides, nitrides and borides reconstruct under OER potentials into amorphous or poorly crystalline oxyhydroxide shells, whereas the reduced precursor may retain HER activity. Thus, the key design variable is no longer only the pristine phase but also its reconstruction pathway and stability [83,84,85]. For example, You et al. [86] utilized machine learning potentials and molecular dynamics simulations to reveal lithium redistribution and dendrite growth governed by grain boundaries in garnet Li_7_La_3_Zr_2_O_12_ and proposed a strategy of in situ grain boundary amorphization under service conditions to suppress lithium aggregation and interfacial protrusions, even with a slight sacrifice of ionic conductivity for improved battery safety and cycling stability. Lattice element leaching creates vacancies, and subsequent hydroxyl filling triggers crystalline-to-amorphous transformation. This reconstruction optimizes electronic configuration and exposes abundant active sites to tune catalytic pathways and performance.

The reconstructed amorphous layers possess hydrated defective metal–oxygen networks with high-valence active centers and fast proton transfer pathways, while the crystalline precursor provides conductive and mechanical support for stable cycling. Operando Raman and XAS are essential to capture transient working-state structural evolution, as post-cycle characterization cannot fully reflect dynamic amorphization processes [87]. In battery systems, long-term cycling induces partial amorphization of crystalline electrodes, improving ion accessibility but potentially causing capacity attenuation, requiring precise structural regulation to balance advantages and defects [88].

### 4.6. Amorphous/Crystalline Hybridization

Amorphous/crystalline hybrid structures integrate the respective strengths of two phases: amorphous domains provide rich unsaturated active sites, flexible coordination and strain buffering; crystalline domains guarantee electronic conductivity, structural robustness and mechanical stability. Three mainstream structural architectures are widely adopted.

First, core–shell structures with amorphous shells coating crystalline cores. Catalyst precursors (phosphides/sulfides) reconstruct into amorphous oxyhydroxide shells during operation, forming conductive core–active shell configurations. Surface-amorphized TiO_2_ nanocrystals represent a classic battery electrode design, balancing fast Li^+^ transport and structural integrity [89]. Second, amorphous/crystalline heterointerfaces regulate interfacial charge transfer and adsorption energy, solving the low conductivity defect of pure amorphous materials [28,74]. Third, amorphous domain embedded nanocomposites disperse disordered motifs in crystalline/conductive matrices, improving ion accessibility and buffering volume change for battery electrodes and supercapacitors [63,72,90]. For example, Xiao et al. [91] constructed Sn-doped CoNiS multipods with innovative amorphous/crystalline interwoven heterostructures, whose abundant phase interfaces modulate the electronic environment of metal sites to optimize polysulfide adsorption and reduce conversion energy barriers, enabling outstanding wide-temperature cycling performance for sodium–sulfur batteries (Figure 4e).

Hybrid structures compensate for the low conductivity and poor reproducibility of pure amorphous materials while increasing structural complexity. High-precision characterization, including HRTEM, STEM-EELS and XPS depth profiling, is required to distinguish interfacial synergistic effects from simple physical mixing. Future synthesis research should focus on amorphous domain spatial distribution, interfacial connection modes and operando reconstruction behavior. A comprehensive comparison of six mainstream synthesis strategies for amorphous energy materials, including their core reaction mechanisms, representative products, prominent advantages and intrinsic defects, is provided in Table 4.

## 5. Characterization, Structural Modeling and Reporting Standards

**Figure 5:**
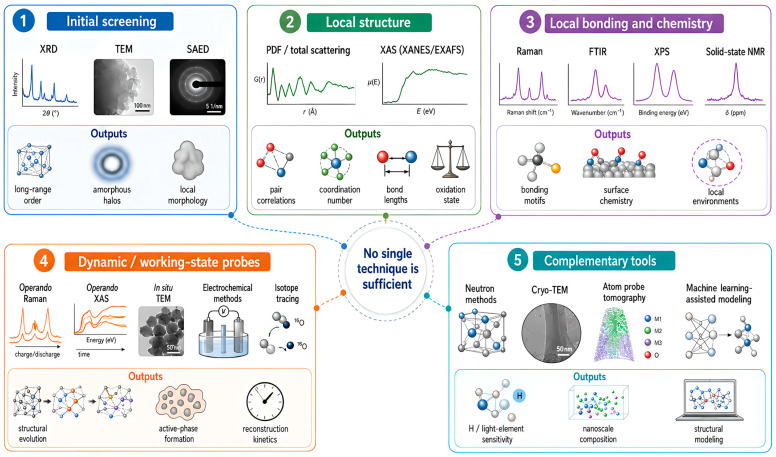
Multimodal characterization workflow for amorphous energy materials. Reliable structure assignment requires long-range screening, local structure analysis, surface/bonding probes, operando working-state detection and experiment-constrained modeling. During the preparation of this figure, the authors used OpenAI ChatGPT (version 5.5) to assist in generating the initial visual layout and graphical elements. The authors reviewed, revised, and scientifically validated all graphical contents and take full responsibility for the final figure.

Characterization is the main bottleneck in amorphous energy material research. Because long-range periodicity is absent, conventional crystallography cannot define the local structures, buried interfaces or electrochemically generated phases that control function. Characterization must therefore move from static phase identification to multi-scale analysis combining diffraction, real-space correlations, spectroscopy, microscopy, electrochemical diagnosis and atomistic modeling [92,93,94,95]. The practical question is not only whether a material is amorphous, but what local motifs exist, where disorder is located and how these features evolve under realistic conditions (Figure 5).

### 5.1. Preliminary Screening: XRD, TEM and SAED

**Figure 6:**
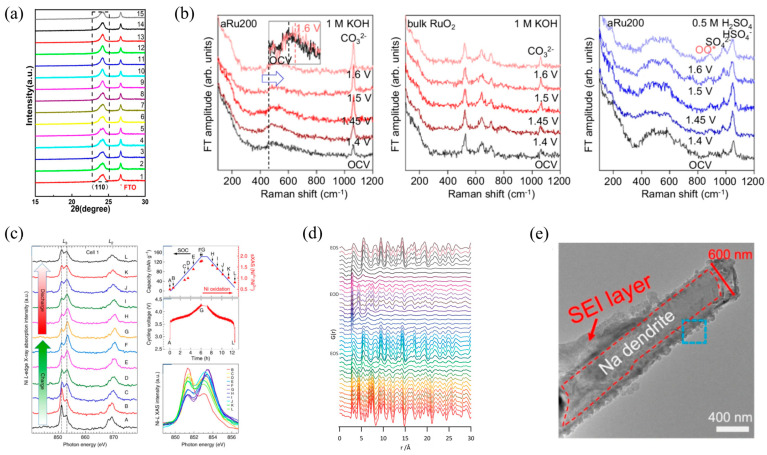
(**a**) Enlarged in situ XRD patterns of the WO_3−x_ NWNs/FTO electrode at 2θ from 15° to 30°. Adapted from Ref. [98]. (**b**) In situ Raman data in 0.5 M H_2_SO_4_ electrolytes. Adapted from Ref. [104]. (**c**) In situ XAS and electrochemical characterizations of the Cell 1 NMC cathode. Adapted from Ref. [110]. (**d**) Selected PDFs obtained during the first discharge, first charge and second discharge cycles by Fourier transforming the total scattering X-ray data. Adapted from Ref. [111]. (**e**) Cryo-TEM of the Na dendrite at a low magnification. Adapted from Ref. [118].

XRD is the most conventional primary screening tool for long-range crystallinity loss, with broad diffraction halos indicating weakened periodicity in amorphous oxides, sulfides and glassy electrolytes. However, ultra-small nanocrystals, highly strained lattices and surface-amorphized particles also produce broad XRD signals, making XRD insufficient for definitive amorphous identification [96,97]. In contrast, time-resolved in situ synchrotron XRD can dynamically track real-time structural evolution under working electrochemical conditions and distinguish transient nonequilibrium intermediate phases hidden from static ex situ XRD measurements. Wang et al. [98] adopted in situ XRD combined with operando spectrum-electrochemical characterizations to unravel the synergistic Al^3+^ intercalation pseudocapacitance mechanism of solvothermally synthesized 3D WO_3−x_ nanowire bifunctional electrodes and constructed electrochromic supercapacitors supporting real-time visual energy readout (Figure 6a).

TEM and SAED provide nanoscale morphological and structural information, with diffuse SAED rings and absent lattice fringes serving as auxiliary amorphous evidence. Their limitations include limited sampling regions, electron-beam-induced structural damage to sensitive amorphous materials, and omission of embedded tiny crystalline domains [99,100]. For amorphous/crystalline hybrid and surface-reconstructed materials, bulk XRD often shows crystalline signals, while surface-sensitive microscopy and spectroscopy detect amorphous surface layers. Thus, preliminary screening requires the integration of synchrotron XRD, statistical HRTEM and SAED analysis.

### 5.2. Local Structure Analysis: PDF/Total Scattering and XAS

PDF analysis based on total scattering converts diffraction data into real-space atomic pair correlation information, revealing atomic spacing, coordination motifs and medium-range structural correlations of amorphous materials [92,101]. It verifies retained short-range structural units in disordered systems and tracks operando structural evolution during battery ion insertion and catalytic reactions, even when crystalline diffraction peaks disappear. Three mainstream total-scattering techniques supply PDF datasets for distinct samples:

High-energy synchrotron X-ray diffraction (HEXRD)

The standard bulk PDF method. Short-wavelength high-energy X-rays deliver broad $Q_{\max}$ for superior real-space resolution and deep penetration to test powders, dense pellets, electrodes and operando devices. X-ray PDF strongly detects heavy transition metals in oxides/sulfides/multi-component electrocatalysts yet offers weak contrast for light elements (H, Li, and O) coexisting with heavy atoms.

Grazing-incidence total scattering (GIPDF/GIXRD)

Tailored for thin films, supported catalysts, surface-amorphized particles and reconstructed interfaces. Near-critical incident angles amplify surface/film scattering while suppressing substrate background. Unlike conventional GIXRD for phase and texture identification, GIPDF covers wide Q ranges to resolve local coordination in seemingly amorphous thin films, though precise corrections for substrate scattering, beam footprint, absorption and grazing geometry are mandatory for quantitative analysis.

Complementary to X-ray PDF, as neutron scattering lengths are uncorrelated with atomic number. It achieves high sensitivity to light elements and differentiates adjacent elements/isotopes inaccessible to X-rays, ideal for Li-bearing electrodes, hydrous oxides and proton conductors where light atoms dominate bonding and ion transport. Neutrons penetrate deeply for bulk characterization under working conditions, but drawbacks include large sample demand, limited beamline access and inferior time resolution compared to synchrotron X-rays.

The method should therefore be selected according to the structural question. HEXRD-PDF is generally preferred for bulk powders, pellets and operando electrochemical cells; GIPDF is preferred for thin films, coatings and near-surface amorphous layers; and neutron PDF is particularly valuable when light elements, isotope contrast or bulk ion distributions are central. Joint X-ray and neutron PDF measurements can provide complementary pair-weighting information and reduce uncertainty in multi-component structural models.

For amorphous energy materials, PDF analysis can determine whether local building blocks persist after the loss of long-range periodicity and can estimate the length scale over which structural correlations decay. It is also suitable for monitoring ion-insertion-induced amorphization, conversion reactions and catalyst reconstruction under working conditions. However, overlapping pair correlations, finite particle size and structural heterogeneity can make PDF models non-unique. PDF results should therefore be constrained by complementary information from XAS rather than interpreted as a complete structural solution on their own.

XAS is an element-selective, crystallinity-independent technique for local structure characterization. XANES spectra reflect oxidation state and local symmetry, while EXAFS calculates coordination number, bond length and structural disorder [51,102]. In OER catalysis, operando XAS identifies valence variation and distorted Fe–O/Co–O active coordination in amorphous catalysts [25,28,103]. In HER research, it confirms terminal disulfide participation in proton reduction of amorphous MoS_x_ [28].

PDF and XAS are highly complementary: PDF provides global atomic correlation information, while XAS delivers element-specific local structural details, jointly defining amorphous short-range order and electrochemical structural evolution.

### 5.3. Local Bonding and Chemical Analysis: Raman, FTIR, XPS and Solid-State NMR

Spectral techniques characterize local bonding, surface chemistry and molecular environments undetectable by diffraction methods. Raman spectroscopy identifies metal–oxygen, metal–sulfur and carbon-based vibration modes, with peak broadening reflecting amorphous structural disorder. Jin et al. [104] employed multiple in situ spectroscopic characterizations centered on in situ Raman analysis to disclose that atomic-scale disorder/amorphization of ultrathin RuO_2_ nanosheets triggers pH-dependent OER mechanisms via distinct lattice oxygen and hydroxyl participation pathways, realizing universal high electrocatalytic activity across acid and alkaline media (Figure 6b).

FTIR supplements functional group and intermediate vibration information, suitable for hydroxyl-containing amorphous oxides, phosphate materials and electrolyte decomposition product analysis in CO_2_RR and battery systems [105,106]. XPS analyzes surface composition, oxidation state and interfacial bonding, tracking post-activation elemental transformation and valence evolution of phosphide-/sulfide-based catalysts [37,85], with depth profiling and operando modes eliminating air exposure interference.

Solid-state NMR detects local environments of Li, Na, P and other nuclei in amorphous electrolytes and battery interphases, explaining ion transport pathways and interfacial stability [58,59,60]. It also captures inserted ions and SEI components undetectable by diffraction methods, complementing microscopic and scattering characterization [107,108].

### 5.4. Dynamic Working-State Detection: Operando Spectroscopy, In Situ TEM and Electrochemical Methods

Amorphous energy materials are dynamically evolving systems, with synthesized structures often differing from real working active phases. Operando characterization is therefore essential to capture electrochemical-induced structural reconstruction.

Operando Raman monitors real-time surface reconstruction and intermediate evolution during electrocatalysis, with simple cell compatibility and high surface sensitivity. Operando XAS directly tracks metal valence, coordination and bond length changes under potential bias, identifying high-activity active structures in catalysts and disorder-induced electrode structural evolution in batteries [109]. For example, Liu et al. [110] developed an operando soft XAS methodology for lithium-ion cathode research and realized real-time Ni valence monitoring of NMC cathode via in situ XAS coupled with electrochemical tests (Figure 6c). Allan et al. [111] employed operando pair distribution function (PDF) analysis combined with ex situ ^23^Na MAS ssNMR spectroscopy to elucidate the stepwise sodiation/desodiation mechanism of Sb anode materials (Figure 6d).

In situ TEM provides high-resolution visualization of morphological evolution, particle cracking and interfacial changes, directly observing lithiation-induced amorphization and conversion reactions [112]. Electrochemical methods (CV, Tafel, EIS, and cycling tests) correlate structural evolution with kinetic performance, distinguishing beneficial activation and harmful degradation. Isotope tracing eliminates false-positive results and clarifies reaction mechanisms for complex amorphous catalytic systems [113,114].

### 5.5. Auxiliary Technologies and Structural Modeling

Neutron scattering compensates for X-ray detection deficiencies in light element analysis, improving structural accuracy of hydrated amorphous oxides and glassy electrolytes [115,116]. Cryo-TEM protects beam-sensitive amorphous phases and fragile battery interphases from electron-beam damage [112,117]. Han et al. [118] adopted cryo-TEM to unveil how fluoroethylene carbonate additives modulate the solid electrolyte interphase architecture on Na metal anodes, demonstrating that a NaF-enriched layered interphase can stabilize the sodium metal/electrolyte interface (Figure 6e). Atom probe tomography (APT) achieves near-atomic-resolution 3D component mapping, revealing nanoscale chemical heterogeneity and interfacial gradients in hybrid amorphous materials [119].

Structural modeling is indispensable for quantitative analysis of amorphous materials. Reverse Monte Carlo, molecular dynamics and DFT simulations construct atomic configurations, simulate ion transport and calculate electronic/adsorption properties [95,96]. Unlike crystalline single-site calculation, amorphous modeling requires statistical sampling of diverse local environments. Machine learning potentials and data-driven spectral analysis rapidly advance, assisting in structural motif identification and structure–performance correlation establishment [95,120].

A practical reporting workflow should include: (i) screening long-range order and morphology by XRD, SAED and statistical TEM; (ii) quantifying local coordination and medium-range order by PDF and XAS; (iii) identifying bonding, surface chemistry and mobile-ion environments by Raman/FTIR, XPS and NMR; (iv) tracking working-state reconstruction by operando Raman, XAS, PDF, TEM or impedance spectroscopy; and (v) constraining atomistic models with experimental descriptors. This workflow should be the minimum standard for amorphous structure–function claims, as shown in Table 5.

### 5.6. Application-Oriented Descriptor Extraction and Standard Analysis Workflow

Structural descriptors are useful only when their measurement, physical meaning, and relationship to electrochemical function are clearly defined. For amorphous materials, most descriptors should be treated as statistical distributions rather than fixed crystallographic constants. Their extraction may also be affected by instrumental resolution, thermal motion, finite particle size, surface contributions, and model selection. A rigorous analysis should therefore begin with a mechanistic question, combine complementary techniques, and compare the pristine, working, relaxed, and aged states. Recent developments in total scattering, operando spectroscopy, cryogenic microscopy, and experiment-constrained modeling provide the methodological basis for such an analysis [92].

#### 5.6.1. Amorphous Electrocatalysts: Coordination and Reconstruction Descriptors

For amorphous NiFe- and Co-based OER catalysts, the primary question is whether catalytic activity originates from the as-prepared material or from a reconstructed oxyhydroxide phase formed during operation. The key descriptors include M–O and M–M coordination numbers, average bond lengths, bond-length variance, metal oxidation state, local symmetry, and reconstruction onset potential.

XRD and statistical TEM/SAED should first determine whether detectable crystalline domains remain. PDF analysis can then quantify local M–O and M–M correlations. The first-shell peak position represents the average nearest-neighbor distance, whereas its width reflects the distribution of bond lengths after instrumental and thermal contributions are considered. The decay of PDF correlations with increasing radial distance provides an operational estimate of structural correlation length.

XANES edge shifts can be calibrated against reference compounds to estimate average oxidation states, while EXAFS fitting provides coordination numbers, bond lengths, and the Debye–Waller factor, σ^2^. Because σ^2^ contains both static disorder and thermal vibration, temperature-dependent measurements are preferred when these contributions must be separated.

Operando measurements should be collected before activation, across the relevant redox region, under steady catalytic turnover, and after potential removal. The reconstruction onset potential may be defined as the potential at which a reproducible XANES shift, EXAFS coordination change, or oxyhydroxide-related Raman band first appears. This value should be compared with the electrochemical redox feature and catalytic-current onset. A convincing structure–activity relationship requires structural reconstruction and catalytic activation to occur over the same potential or time range.

For amorphous MoSx HER catalysts, relevant descriptors include Mo–S coordination, Mo valence, terminal-to-bridging sulfur ratio, and electrochemical activation state. XAS provides average Mo–S coordination, whereas Raman and XPS help distinguish terminal sulfide, bridging sulfide, and disulfide-like species. A sulfur motif should be regarded as catalytically relevant only when its population changes reproducibly during activation and correlates with overpotential, Tafel slope, or charge-transfer resistance [37].

#### 5.6.2. Amorphous Battery Electrodes: Reversible Disorder Versus Degradation

For amorphous vanadium oxides, surface-amorphized TiO_2_, and Si-based anodes, the central objective is to determine whether structural disorder improves ion transport and strain accommodation or represents irreversible collapse. Relevant descriptors include PDF correlation length, mobile-ion coordination, local redox reversibility, free volume, pathway connectivity, strain evolution, and impedance growth [42].

Operando XRD should first track the displacement, broadening, disappearance, or recovery of Bragg reflections. However, peak disappearance alone does not demonstrate complete amorphization. Operando total scattering and PDF analysis should follow characteristic metal–oxygen, metal–metal, and metal–anion correlations after long-range order is lost. Reversible changes in peak position and width indicate local bond rearrangement, whereas permanent damping of medium-range correlations suggests irreversible structural degradation.

XAS measurements at selected states of charge can identify the redox-active element and determine whether its coordination environment recovers after cycling. Reversible XANES shifts accompanied by recovery of EXAFS coordination and bond length support reversible local redox. Incomplete valence recovery, continuous coordination loss, or progressive growth of σ^2^ may indicate trapped ions, conversion-product accumulation, or irreversible rearrangement.

Solid-state NMR can resolve Li^+^, Na^+^, or other mobile-ion environments that are invisible to diffraction. Chemical shifts distinguish different sites, integrated peak areas estimate their populations, and relaxation or exchange measurements probe local dynamics. Broad NMR signals should not automatically be interpreted as fast ion transport because static disorder, chemical heterogeneity, and paramagnetic interactions can produce similar broadening.

Free volume may be evaluated using positron-annihilation lifetime spectroscopy, density measurements, scattering-constrained atomistic models, or molecular dynamics. Gas-accessible porosity should not be treated as equivalent to the subnanometer free volume controlling ion hopping. Free-volume descriptors should therefore be correlated with diffusion coefficients, activation barriers, rate capability, and impedance rather than interpreted independently.

Beneficial amorphization is supported when local redox motifs remain reversible, ion mobility improves, mechanical strain is accommodated, and impedance remains stable. Conversely, disorder accompanied by persistent voltage hysteresis, trapped-ion signals, loss of redox reversibility, or continuous impedance growth should be classified as degradation.

#### 5.6.3. Hydrous Pseudocapacitive Materials: Hydration and Near-Surface Redox

For hydrous amorphous MnO_2_, RuO·xH_2_O, and transition-metal hydroxides, charge storage is strongly affected by structural water, local M–O coordination, ion–solvent exchange, and near-surface redox. The main descriptors include total and coordinated water contents, metal valence, M–O coordination, reversible mass change, ion–solvent uptake, and charge-transfer resistance [69].

Thermogravimetric analysis, preferably coupled with mass spectrometry or infrared gas analysis, can quantify water release and distinguish it from ligand or electrolyte decomposition. Raman, FTIR, solid-state NMR, or neutron scattering should then be used to differentiate weakly adsorbed water from structurally coordinated water.

Operando XAS can track changes in metal valence, M–O bond length, and coordination number during charging, while operando Raman identifies reversible changes in local bonding and hydration. Electrochemical quartz crystal microbalance measurements relate stored charge to mass variation and can help distinguish proton transfer, hydrated-cation insertion, anion adsorption, and solvent co-insertion. Viscoelastic changes and trapped solvent must be considered before applying the Sauerbrey relation.

Impedance spectroscopy should monitor charge-transfer resistance and diffusion-related processes throughout cycling. Capacitance retention alone is insufficient to establish structural stability; a robust assessment should demonstrate reversible mass and coordination changes, stable hydration-assisted transport, limited dissolution, and no continuous impedance growth.

#### 5.6.4. Amorphous Solid Electrolytes and Interphases

For LiPON-related materials, sulfide glasses, amorphous halides, and electrode–electrolyte interphases, useful descriptors include network-former connectivity, mobile-ion coordination, site population, bottleneck distribution, activation energy, interphase thickness, and interfacial resistance.

X-ray and neutron total scattering should be combined where possible because their different pair sensitivities improve the resolution of light-element environments. PDF analysis and experiment-constrained structural models can quantify characteristic bond distances, coordination numbers, and the connectivity of network-forming units. Solid-state NMR can distinguish Li, Na, P, B, Si, S, or halogen environments and quantify their relative populations. Variable-temperature relaxation and exchange measurements can separate immobile sites from dynamically exchanging ion populations [87].

Bulk, grain-boundary, and interfacial impedance contributions should be distinguished using characteristic capacitance, frequency range, sample-geometry dependence, and temperature response. Equivalent circuits must be physically justified, while distribution-of-relaxation-time analysis can help separate overlapping processes.

For artificial amorphous interphases, thickness and composition should be measured using cryo-TEM/EELS, depth-resolved XPS, or secondary-ion mass spectrometry. A beneficial interphase should be thin, chemically self-limiting, ionically conductive, and electronically insulating. Its function must be demonstrated by correlating interphase formation with stable or reduced interfacial resistance rather than inferred from microscopy alone.

#### 5.6.5. Recommended Standard Workflow

A standardized analysis of amorphous and disordered energy materials should follow this sequence:

1. Define the mechanistic question. Specify whether the study addresses active-site formation, ion transport, hydration-assisted storage, strain accommodation, interphase stabilization, or degradation.
2. Define the material state. Clearly distinguish the as-synthesized, activated, operating, relaxed, discharged, and aged states.
3. Select suitable controls. Include compositionally matched crystalline or poorly crystalline references and measure contributions from substrates, binders, conductive additives, electrolytes, and operando cells.
4. Screen long-range order. Combine XRD, SAED, and statistically representative TEM. Report detection limits, sampling areas, and possible beam-induced transformations.
5. Quantify local and medium-range structure. Extract coordination numbers, bond lengths, bond-length variances, PDF peak widths, and correlation lengths using consistent fitting procedures.
6. Determine local chemistry. Use XANES, XPS, Raman, FTIR, EELS, and NMR to identify oxidation states, bonding motifs, structural water, mobile-ion sites, and interphase products.
7. Track working-state evolution. Perform operando measurements at electrochemically meaningful potentials, states of charge, temperatures, or gas compositions.
8. Correlate descriptors with performance. Align structural changes with overpotential, Tafel slope, Faradaic efficiency, capacity, voltage hysteresis, conductivity, impedance, and degradation rate.
9. Assess reversibility. Compare pristine, working, relaxed, and aged states to distinguish adaptive reconstruction from irreversible degradation.
10. Constrain structural models experimentally. RMC, molecular dynamics, DFT, and machine learning models should reproduce multiple independent observables rather than a single PDF or XAS dataset.
11. Report uncertainty. Provide fitting ranges, background treatment, calibration standards, parameter covariance, confidence intervals, sampling statistics, and sensitivity to alternative models.

This workflow moves characterization beyond qualitative claims that disorder improves electrochemical performance. It establishes a reproducible chain from measurement and descriptor extraction to working-state mechanism and performance correlation, thereby enabling meaningful comparison among amorphous materials prepared by different routes or used in different electrochemical systems.

## 6. Applications in Electrochemical Energy Conversion

Amorphous energy materials are widely used in electrochemical and photoelectrochemical conversion. These reactions involve coupled proton, electron and ion transfer, where activity depends on local coordination, valence flexibility, adsorption energy distribution and dynamic reconstruction rather than long-range order alone. The main systems include HER/OER water splitting, ORR/OER oxygen electrocatalysis, CO_2_RR, NRR and photocatalytic/PEC conversion [23,34,121].

### 6.1. Water Splitting: HER and OER

Water splitting, including HER and OER, is the most representative application of amorphous electrocatalysts. HER optimizes hydrogen adsorption, proton/electron transfer and H_2_ desorption; OER involves four-electron oxidation, multiple oxygen-containing intermediates and high-valence metal–oxygen species. Mechanistic differences lead to different material design directions: amorphous sulfides, phosphides, borides and alloys are mainly used for HER, while amorphous oxyhydroxides and reconstructed transition-metal compounds dominate OER.

Beyond water electrolysis for energy conversion, amorphous electrocatalysis also underpins electrochemical pollutant remediation, and relevant research deepens fundamental understanding of heterogeneous electrocatalytic reaction pathways. For instance, one representative investigation [122] delivers fundamental new insights into the electrochemical oxidation process for organic pollutant detection via amperometric COD sensing. It elaborates a full electrocatalytic cycle: adsorbed OH^−^ electrochemically converts Cu(II)O into oxidative Cu(III)-·OH intermediates that mineralize organics, regenerating Cu(II)O and generating pollutant-concentration-dependent current signals, while also clarifying how chitosan modification lowers charge-transfer resistance to accelerate interfacial electron transfer and optimize overall electrooxidation kinetics. This body of work universally demonstrates that identifying intermediate evolution, interfacial charge transport and dynamic structural reconstruction is the core to unraveling electrocatalytic mechanisms across energy conversion and pollutant degradation systems.

Crystalline MoS_2_ has obvious anisotropy: basal planes are inert and edge sites are active for HER [123]. Amorphous MoS_x_ without layered periodicity exposes a large number of unsaturated Mo and S sites, getting rid of the limitation of crystal edge density. This structural advantage is very suitable for thin-film electrodes and photoelectrochemical photocathodes requiring conformal catalyst coating and tight contact with light absorbers.

**Figure 7:**
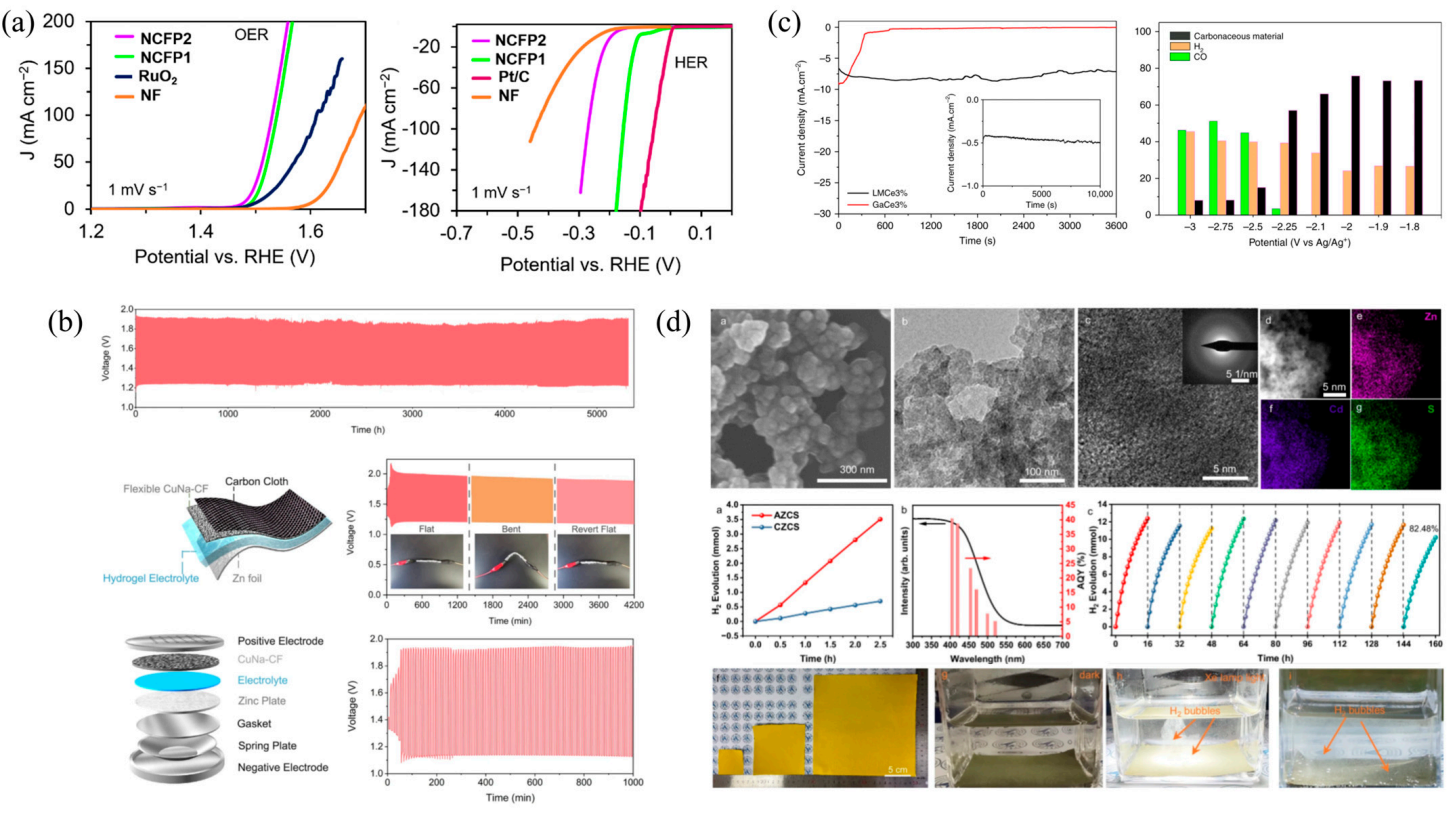
(**a**) The LSV curves for the OER and HER activities of the NCFP catalysts. Adapted from Ref. [124]. (**b**) The liquid zinc–air battery cycling performance of the CuNa-CF air cathode and schematics of flexible and button-type all-solid-state zinc–air batteries, together with cycling operational stability and digital photographs of the flexible device under different bending states. Adapted from Ref. [129]. (**c**) Chrono-amperometry results for liquid galinstan and solid gallium, and Faradaic efficiencies of LMCe3% for the production of CO and H_2_. Adapted from Ref. [133]. (**d**) Ambient electrocatalytic NRR performance of BVC-A, including NH_3_ yield, Faradaic efficiency, cycling stability, catalyst comparison and corresponding UV–Vis spectra. Adapted from Ref. [135].

For OER, amorphous cobalt phosphate and NiFe oxyhydroxides are classic research systems. The CoPi catalyst is formed in situ from Co^2+^-containing phosphate electrolytes under anodic potential and works as a self-assembled amorphous catalyst in near-neutral electrolytes [66]. Mechanistic studies prove that cobalt–oxygen networks, proton-coupled electron transfer and the phosphate buffer effect jointly control OER performance [43]. This work verifies that high-efficiency OER active phases can be dynamically generated under electrochemical working conditions. Moloudi et al. [124] synthesized amorphous Ni–Co–Fe–P via one-pot potential pulse electrosynthesis, which exhibits outstanding trifunctional electrocatalysis for HER, OER and ORR and achieves remarkable performance in alkaline seawater electrolysis and zinc–air batteries under industrial high mass loadings (Figure 7a).

Corrigan first found that trace Fe impurities significantly improve the OER activity of Ni oxide electrodes [125]. Follow-up research confirms that intentional and unintentional Fe doping determines the performance of NiFe oxyhydroxide catalysts [45]. Operando XAS identifies Fe sites with unique local coordination as high-activity OER centers, proving that the local Fe–O environment and high-valence metal–oxygen structure dominate catalytic performance [126]. Amorphous and poorly crystalline oxyhydroxide networks adapt to high-valence metal centers and distorted M–O coordination that are difficult to form in crystalline lattices.

Transition-metal phosphides, sulfides, nitrides, carbides and borides are universal water-splitting catalysts. Most of them have good HER activity in the reduced state, but their surfaces reconstruct into amorphous/poorly crystalline oxyhydroxide layers under anodic OER potential [85]. Taking Ni_2_P as an example, its OER activity originates from surface oxidation and oxyhydroxide formation rather than the original phosphide phase [127]. Most amorphous OER catalysts are essentially working-state reconstructed materials: the amorphous oxyhydroxide shell acts as the active phase, and the crystalline/metallic core provides conductivity and mechanical support.

In water-splitting reactions, amorphous materials adopt different working mechanisms for HER and OER: amorphous sulfides/phosphides expose unsaturated active sites and adjust hydrogen adsorption for HER; amorphous oxyhydroxides stabilize high-valence M–O motifs and realize continuous dynamic reconstruction for OER. High-quality research needs to combine electrochemical performance with operando Raman, XAS, isotope tracing and post-operation microscopy to confirm real active phases.

### 6.2. Oxygen Electrocatalysis for Metal–Air Batteries and Fuel Cells

Oxygen electrocatalysis, including ORR and OER, is the core of fuel cells, rechargeable metal–air batteries and regenerative electrochemical systems. In rechargeable Zn–air batteries, ORR proceeds during discharge and OER during charge, so air electrodes require bifunctional catalytic activity, high electronic conductivity, smooth gas diffusion, electrolyte compatibility and long-term stability. Amorphous oxides, hydroxides, carbon-supported metal sites and amorphous/crystalline hybrid materials integrate flexible adsorption environment and interfacial charge transport, becoming ideal oxygen electrocatalysts.

Stable electronic conductivity and appropriate O_2_ adsorption are the key requirements for ORR. Nitrogen-doped carbon, Fe–N–C and Co–N–C materials are alternatives to precious Pt-based catalysts. Most M–N–C catalysts contain graphitic/turbostratic carbon domains, and their active sites are locally disordered and embedded in amorphous/partially graphitized carbon matrices. Nitrogen-doped carbon nanotube arrays show better durability and methanol tolerance than Pt/C catalysts for ORR [128]. Nevertheless, conventional M–N–C catalysts still suffer from unsatisfactory catalytic stability under long-term battery cycling, which can be effectively ameliorated by introducing adjacent main-group metal functional groups to modulate the electronic structure of metal active centers. Li et al. [129] synthesized flexible self-supporting Cu/Na dual-atom carbon membranes via blow spinning–pyrolysis. Neighboring Na species activate and stabilize Cu-N_4_ sites, endowing liquid and flexible solid zinc–air batteries with ultra-long cycling durability exceeding 5000 h for practical battery development (Figure 7b).

Amorphous manganese oxides and bimetallic oxides are important oxide-based oxygen electrocatalysts. Mn, Co, Ni and Fe have variable oxidation states and flexible oxygen coordination environments. Amorphous MnO_x_ loaded on conductive substrates acts as a bifunctional ORR/OER catalyst for Zn–air batteries, with abundant surface defects and mixed Mn valence contributing to oxygen electrocatalysis. Amorphous bimetallic oxide–graphene hybrids combine disordered oxide active sites and conductive graphene networks to optimize bifunctional performance.

The performance requirements of ORR and OER are different: ORR needs stable, high-conductivity and relatively fixed active sites; OER operates under high anodic potential and is accompanied by surface oxidation and catalyst reconstruction. Therefore, high-efficiency bifunctional catalysts mostly adopt hybrid structures: carbon/crystalline conductive frameworks undertake ORR and electron transport, and amorphous/reconstructable metal oxyhydroxide surfaces are responsible for OER. This design promotes the research of amorphous/crystalline composite catalysts, core–shell structures and reconstructed air electrodes for metal–air batteries [130].

### 6.3. CO_2_ Reduction and Nitrogen-Related Electrocatalysis

CO_2_RR converts CO_2_ into CO, formate, methanol, methane, ethylene and other high-value chemicals, with strict selectivity requirements. Multiple products compete in a narrow potential window, and the binding energy of intermediates such as ·CO_2_^−^, ·COOH, ·OCHO and ·CO determines product distribution [131]. Amorphous materials have heterogeneous active site ensembles and flexible binding environments, stabilizing different intermediates in multi-step reaction pathways and regulating product selectivity.

Bi-based, Sn-based and In-based oxides and oxide-derived catalysts are widely studied for formate production. These catalysts undergo cathodic reduction, surface roughening and reconstruction during CO_2_RR, forming locally disordered/amorphous surfaces that favor ·OCHO intermediate formation. The as-prepared material acts as a precursor, and the reconstructed working-state surface controls catalytic performance [106,132]. For instance, Esrafilzadeh et al. [133] developed liquid metal electrocatalysts embedded with Ce-based amorphous interfacial oxides for room-temperature CO_2_ electroreduction into solid carbon products, where the amorphous oxide species synergize with Ce nanoparticles to lower reaction onset potential and suppress coking deactivation, realizing recyclable negative carbon emission technology with carbon products applicable to supercapacitors (Figure 7c).

Carbon-supported and molecular-derived catalysts have amorphous-like structural characteristics. Atomically dispersed Ni–N–C and Fe–N–C catalysts for CO_2_-to-CO conversion have local metal–nitrogen coordination embedded in disordered carbon matrices. These materials do not belong to traditional amorphous glasses, but their active environments have no long-range periodicity and are characterized by local coordination descriptors. Disordered carbon adjusts electronic density, local hydrophobicity and CO_2_ accessibility, and inhibits competitive HER.

Defect-rich carbon, amorphous metal oxides, sulfides and single-atom catalysts are applied to NRR because N_2_ activation requires strong local polarization and electron supply. The research of NRR catalysts needs to strictly eliminate ammonia pollution, nitrate/nitrite impurities and background signal interference, and ^15^N_2_ isotope tracing and pollution control are essential. Lv et al. [134] developed amorphous Bi_4_V_2_O_11_/CeO_2_ hybrid electrocatalysts for ambient NRR, where CeO_2_ induces amorphous defective active sites and constructs matched band alignment for fast interfacial charge transfer, delivering a much superior ammonia yield and Faradaic efficiency compared to its crystalline counterpart (Figure 7d).

For carbon/nitrogen-related electrocatalysis, the advantage of amorphous structures is reflected in flexible intermediate binding, variable local valence and diverse active site distribution, rather than simply high specific surface area. The complexity of active sites increases the difficulty of mechanism research, so selectivity-oriented operando spectroscopy, isotope labeling and quantitative product analysis are necessary for standard research.

### 6.4. Photocatalysis and Photoelectrochemical Conversion

In photocatalysis and photoelectrochemical (PEC) conversion, amorphous materials are used as passivation layers, co-catalysts, protective coatings and interfacial charge-transfer layers. Different from pure electrocatalysis, PEC systems need to optimize light absorption, charge separation, surface reaction kinetics and semiconductor stability simultaneously. Amorphous layers coat semiconductor surfaces conformally, passivate dangling bonds, inhibit surface charge recombination and provide abundant catalytic active sites.

Amorphous molybdenum sulfide combined with Cu_2_O photocathodes realizes PEC hydrogen production. Amorphous MoS_x_ co-catalysts accelerate HER kinetics on the photocathode surface while maintaining tight contact with light absorbers [135]. Amorphous CoPi and cobalt oxide co-catalysts are deposited on photoanodes such as BiVO_4_, Fe_2_O_3_ and TiO_2_ to promote water oxidation and reduce surface recombination [136,137]. Wang et al. [135] verified that disordered amorphous ZnCdS generates internal electric fields to accelerate charge separation, and Co-MoS_x_-loaded amorphous ZnCdS achieves remarkable durable hydrogen evolution, while its scalable flexible film holds great promise for large-scale solar hydrogen production. These applications define amorphous co-catalysts as functional interfacial reaction layers rather than independent bulk catalysts.

Amorphous TiO_2_, Al_2_O_3_ and NiO_x_ act as protective and passivation coatings for semiconductor photoelectrodes. Semiconductor corrosion and surface recombination are the main limitations of PEC devices. Thin amorphous oxide layers protect light absorbers while allowing charge tunneling and selective carrier transport. Excessively thick or highly insulating amorphous coatings block charge transfer and reduce photocurrent. The key design parameters include component, layer thickness, band alignment, defect density and charge-transfer resistance.

Amorphous materials can introduce mid-gap states to broaden the light absorption range, but excessive disorder increases trap-assisted recombination and reduces carrier mobility. Therefore, amorphous layers in photocatalysis and PEC conversion are designed as functional interfaces: passivating defects, extracting charges and accelerating surface reactions, without introducing additional recombination centers.

## 7. Applications in Electrochemical Energy Storage

In energy storage, amorphous materials mainly improve ion transport and mechanical tolerance. Their disordered frameworks provide isotropic diffusion pathways, abundant storage sites and strain buffering during cycling. They are therefore attractive for large-ion or multivalent systems, conversion and alloying electrodes, pseudocapacitors and solid electrolytes. However, excessive disorder can reduce electronic conductivity, increase side reactions and impair structural reversibility.

### 7.1. Lithium-Ion and Post-Lithium-Ion Batteries

Amorphous materials find broad applications in lithium, sodium and potassium ion batteries. For intercalation-type electrodes, their disordered frameworks deliver isotropic ion diffusion and abundant storage sites, outperforming anisotropic crystalline channels. For conversion and alloying-type electrodes, amorphous structures effectively buffer drastic volume variation and prevent particle pulverization. These merits are especially critical for Na^+^ and K^+^ storage: their large ionic radii and strong solvation effects lead to sluggish ion migration in rigid crystalline lattices, while amorphous architectures resolve this kinetic limitation well.

Surface-amorphized TiO_2_ nanocrystals serve as a typical example. The disordered surface layer accelerates Li^+^ migration and interfacial reaction kinetics, while the crystalline core maintains structural integrity, enabling outstanding high-rate lithium storage performance [89]. This proves that partial amorphization often achieves better comprehensive performance than full amorphization, balancing fast ion transport and mechanical stability.

**Figure 8:**
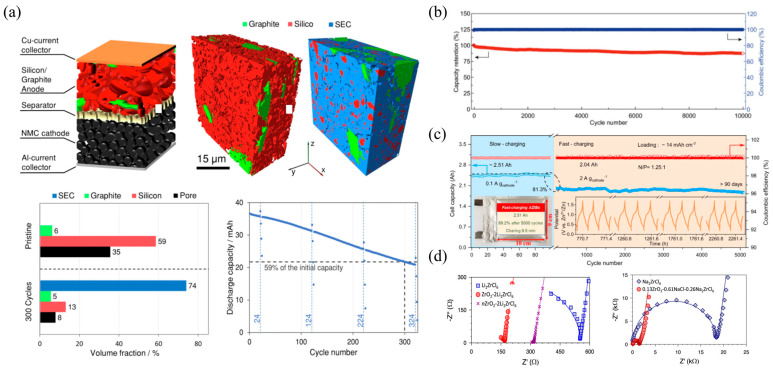
(**a**) Experimental setup of the utilized Si-anode, electrochemical performance and microstructure degradation. Adapted from Ref. [138]. (**b**) Cycling stability of RuO_2_·H_2_O||Zn battery. Adapted from Ref. [145]. (**c**) The capacity and coulombic efficiency of the pouch cells during long cycling. Adapted from Ref. [56]. (**d**) Ionic conductivity curves of halide solid electrolytes. Adapted from Ref. [149].

Amorphous vanadium oxide exhibits superior sodium storage capability compared with crystalline counterparts, owing to its open, flexible disordered framework that creates more accessible Na^+^ accommodation sites and unconstrained diffusion paths [32]. With multiple oxidation states and adjustable V-O coordination, vanadium oxide systems readily adapt to structural rearrangement during repeated charge and discharge cycles. Häusler et al. [138] revealed that lithium-ion permeation induces interfacial amorphization of silicon anodes during lithium insertion/extraction, and the cycling-generated non-hydrostatic strain further triggers internal dislocation and shear band evolution, which causes uneven lithiation, disturbs SEI reconstruction and deteriorates battery capacity (Figure 8a).

Conversion-type electrodes also benefit greatly from amorphization. Nano-sized transition metal oxides realize high lithium storage capacity via reversible conversion reactions and gradually evolve into composite structures of metal nanoparticles dispersed in Li_2_O-based matrices after cycling [139]. The resultant disordered phases buffer volume expansion and sustain reversible redox reactions, operating via mechanisms distinct from conventional intercalation.

Silicon-based anodes are representative alloying-type materials. Crystalline silicon suffers from severe anisotropic volume expansion during lithiation, which easily triggers electrode cracking and unstable solid–electrolyte interphase (SEI) growth. In contrast, amorphous silicon and silicon-based composites uniformly distribute internal stress, suppress crack propagation and improve cycling durability [57,88]. Hard carbon, the dominant anode material for sodium-ion batteries, features turbostratic domains, amorphous regions and abundant pores. Its intrinsic disorder enables Na^+^ storage through adsorption, intercalation and pore filling, making it the mainstream choice for practical sodium-ion batteries [140].

Nevertheless, excessive structural disorder brings unavoidable drawbacks: it lowers electronic conductivity, increases irreversible capacity loss and broadens voltage profiles. To address these issues, practical amorphous electrode materials are commonly engineered with nanocrystallization, conductive carbon networks, surface coatings and electrolyte optimization. These strategies synergistically improve ion accessibility while guaranteeing structural reversibility and long-term stability.

### 7.2. Aqueous Zinc-Ion and Multivalent-Ion Batteries

Aqueous zinc-ion and other multivalent-ion batteries face unique challenges: the high charge density of Zn^2+^, Mg^2+^, Ca^2+^ and Al^3+^ causes strong electrostatic interaction with host frameworks, leading to slow solid-state diffusion and high desolvation barriers. Amorphous and hydrated structures stand out here, as their flexible coordination environments, structural water and disordered diffusion channels effectively reduce kinetic resistance for multivalent ion transport.

MnO_2_-based cathodes are widely studied for aqueous zinc-ion batteries. Rechargeable Zn–MnO_2_ batteries achieve high energy and power density, with energy storage relying on complex proton/Zn^2+^ insertion, phase transition and electrolyte participation [71]. Amorphous or poorly crystalline MnO_x_ phases readily form during cycling, and structural disorder together with bound water facilitates ion migration. Even so, manganese dissolution and irreversible structural evolution remain major obstacles to long-cycle stability.

Vanadium-based oxides are another key category for zinc and multivalent-ion batteries. Hydrated and amorphous vanadate frameworks provide flexible accommodation for Zn^2+^ and coexisting water molecules, supporting dual-ion insertion and high-capacity output [141,142]. Liu et al. [56] designed an amorphous organic-hybrid vanadium oxide cathode with disordered chain structure to drastically cut Zn^2+^ diffusion activation energy, enabling pouch cells with ultra-fast charging and superior cycling stability for high-performance zinc-ion batteries (Figure 8b). However, their flexible structures also raise risks of material dissolution and catastrophic structural collapse under prolonged cycling.

For Mg^2+^, Ca^2+^ and Al^3+^ batteries, amorphous host materials offer adaptable coordination sites and softened diffusion channels to mitigate the strong interaction between multivalent ions and lattice anions. Yet intense ion–lattice coupling may still cause ion trapping, sluggish kinetics and structural degradation. In general, amorphous materials for multivalent-ion batteries rely on hydrated pathways and weakly bonded frameworks to boost performance, while suppressing dissolution and irreversible phase change is the core priority for future development. Operando XRD/PDF, XAS, Raman and post-cycle chemical analysis are essential to distinguish reversible amorphous ion storage from irreversible material degradation.

### 7.3. Supercapacitors

Amorphous oxides, hydroxides, sulfides and carbon materials are ideal candidates for supercapacitors, thanks to their large specific surface area, rich redox sites and shortened ion diffusion lengths. Pseudocapacitance originates from rapid surface or near-surface redox reactions, a process well matched to the flexible local coordination of amorphous structures, which enables fast ion adsorption/desorption and reversible redox switching.

Amorphous MnO_2_ and its carbon nanotube composites are classic pseudocapacitive electrodes. Disordered Mn-O networks provide plentiful accessible redox sites, while carbon additives enhance electronic conductivity and structural stability [143]. As summarized in classic theories, high-rate capacitive performance requires short diffusion paths and efficient charge transfer—advantages inherently possessed by amorphous MnO_2_ systems [144].

Nickel–cobalt hydroxides, nickel hydroxides and cobalt hydroxides in amorphous or poorly crystalline states expose abundant active metal sites and unobstructed OH^-^ transport pathways in alkaline electrolytes. Their main limitations lie in their poor intrinsic conductivity and volume fluctuation during cycling, which are commonly relieved via amorphous/crystalline hybridization, carbon compounding and nanosheet array design.

Hydrous amorphous RuO_2_ is a benchmark high-performance pseudocapacitive material. Its excellent capacitance stems from fast proton–electron transport within hydrated amorphous networks. Dong et al. [145] first adopted amorphous RuO_2_·H_2_O as a cathode for aqueous zinc-ion hybrid capacitors, realizing ultra-fast Zn^2+^ pseudocapacitive storage with outstanding energy/power densities and a lifespan exceeding 10,000 cycles to advance safe high-power energy storage devices (Figure 8c). Though ruthenium-based materials are costly, the working mechanism has guided the development of low-cost alternatives, including Mn, Ni, Co and Fe-based amorphous oxides and hydroxides.

For carbon-based supercapacitors, amorphous and disordered carbon delivers a large surface area and diverse pore structures. Charge storage mainly follows the electric double-layer mechanism, while heteroatom doping (O, N, S, and P) introduces additional pseudocapacitance. The core design principle is to balance micropores for high capacitance and meso-/macropores for rapid ion transport.

In supercapacitor systems, repeated redox cycling may alter material hydration state, local bonding and pore structure. Thus, stability evaluation should cover both capacitance retention and the persistence of local coordination, hydrated environments and conductive networks. Operando Raman, electrochemical quartz crystal microbalance, XAS and impedance spectroscopy can effectively monitor ion insertion, hydration variation and charge-transfer behavior.

### 7.4. Solid-State Electrolytes and Ion Conductors

Amorphous and glassy ion conductors are vital components for solid-state electrochemical devices, as their disordered atomic networks establish continuous ion transport channels, alleviate grain-boundary lattice mismatch, and strengthen electrode–electrolyte interfacial contact. The practical performance of these materials relies on a comprehensive set of merits beyond bulk ionic conductivity, including facile processability, wide chemical compatibility, favorable mechanical flexibility, and stable interphase evolution.

For room-temperature all-solid-state batteries, amorphous LiPON stands as the benchmark thin-film solid electrolyte. Despite its moderate ionic conductivity relative to sulfide counterparts, LiPON demonstrates outstanding chemical inertness against metallic lithium, supporting stable long-term cycling in thin-film microbatteries and highlighting that interfacial compatibility carries equal weight to intrinsic bulk conductivity [146,147]. Its success proves that interfacial compatibility is equally important as bulk conductivity for solid electrolytes.

Meanwhile, Li_2_S–P_2_S_5_ sulfide glasses and glass–ceramics deliver superior Li^+^ conductivity and plastic deformability for tight electrode lamination; though crystalline LGPS electrolytes achieve record-high conductivity, amorphous sulfides remain competitive via scalable fabrication and compliant interfaces [148]. A critical drawback of sulfide systems, however, is severe oxidative decomposition at high potentials, restricting pairing with high-voltage layered oxide cathodes. To address this bottleneck, Kwak et al. [149] fabricated ZrO_2_-modified halide nanocomposite solid electrolytes with boosted Li^+^/Na^+^ ionic conductivity and enhanced interfacial compatibility, and the fluorinated electrolyte delivers excellent high-voltage stability, enabling long-cycle all-solid-state batteries paired with layered oxide cathodes (Figure 8d).

Amorphous species exert distinct effects in 600–800 °C solid oxide fuel/electrolysis cells (SOFC/SOEC), which adopt crystalline ionic conductors. Spontaneous amorphous grain-boundary phases from impurity segregation (e.g., Si segregation in YSZ) and thermal interdiffusion hinder oxide-ion migration and raise ohmic resistance, even as discrete nano-sized siliceous domains. Meanwhile, electrode–electrolyte interfaces suffer cation interdiffusion, Sr/Cr segregation and secondary-phase generation, forming resistive layers that boost polarization loss; high-current electrolysis worsens such interfacial degradation. By contrast, rationally fabricated thin amorphous interlayers fill interfacial voids, relieve thermal stress mismatch and suppress cracking/delamination to enhance cell durability. Yet these merits are not inherent to amorphous materials. Functional interlayers require an ultrathin, intact geometry, decent ionic conductivity, negligible electron leakage and high thermal stability against crystallization and element interdiffusion. Mismatched composition or excessive thickness turns such layers into insulative barriers over cycling. Thus, high-temperature amorphous matter falls into two categories: detrimental spontaneously formed impurities that impede ion conduction and tailor-made functional interlayers for interface stabilization. The design priority lies in thin, thermally compatible interfacial films rather than a high amorphous content, which can offset degradation-induced resistance without severe transport loss [1,11,23].

Future studies should combine temperature-dependent impedance spectroscopy with isotope exchange, high-resolution TEM–EELS, atom probe tomography, Raman spectroscopy and depth-resolved chemical analysis. Measurements before operation, during thermal aging and after electrochemical testing are needed to determine whether an interfacial amorphous phase functions as an ion-conducting bridge, a stress-buffering layer or a resistive degradation product. This distinction provides a more rigorous basis for extending amorphous-material concepts from low-temperature batteries to high-temperature SOFCs and SOECs.

## 8. Working-State Structural Evolution and Stability

A key shift in amorphous energy materials is to treat them as dynamically evolving systems rather than fixed structural states. Their structures respond to potential, pH, electrolyte composition, ion insertion, stress and cycling history. Controlled evolution can generate active catalytic phases, conductive ion pathways and strain-resistant frameworks; uncontrolled evolution causes dissolution, over-reconstruction, electrical isolation and collapse [150].

**Figure 9:**
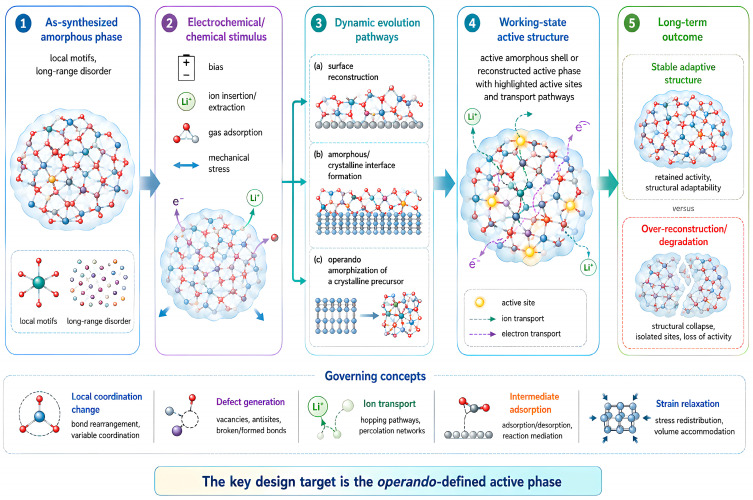
Working-state structural evolution of amorphous energy materials. The sequence links the as-synthesized state, external stimulus, dynamic evolution pathways, operando active structure and long-term performance outcome. During the preparation of this figure, the authors used OpenAI ChatGPT (version 5.5) to assist in generating the initial visual layout and graphical elements. The authors reviewed, revised, and scientifically validated all graphical contents and take full responsibility for the final figure.

The evolution process follows a general sequence (Figure 9): the as-synthesized amorphous or semi-amorphous phase is stimulated by electrochemical, chemical or mechanical factors, causing coordination changes, defect formation, ion migration, adsorption and strain relaxation. These processes generate working-state structures such as amorphous shells, reconstructed oxyhydroxides, ion-conducting interphases and amorphous/crystalline interfaces. Stability should therefore be evaluated by the controllability and function of structural evolution rather than preservation of the initial phase.

### 8.1. From Static Amorphousness to Operando-Defined Active Structures

Traditional research correlates performance with the static structure of as-prepared materials, which is no longer applicable to amorphous energy materials. The pristine structure often differs drastically from the real active phase under working conditions. In electrocatalysis, many crystalline or amorphous precursors reconstruct into new surface phases under applied potential. In batteries, repeated ion insertion and extraction induce amorphization, partial recrystallization and interphase formation. In solid-state batteries, thin initial amorphous interlayers gradually evolve into complex composition-gradient phases, which either stabilize or deteriorate electrode–electrolyte interfaces [150].

This concept is most prominent in OER research. Operating under strong oxidizing potentials, most transition metal compounds undergo surface oxidation, ligand exchange and dissolution–redeposition to form oxyhydroxide layers. Researchers have re-examined the nature of metal chalcogenides, nitrides and phosphides, pointing out that most of them act as precursors rather than intrinsic OER catalysts [150]. Multiple operando studies confirm that the surface layer formed in situ dominates catalytic activity [92].

For amorphous materials, this distinction is even more critical. An amorphous precursor may deliver high activity owing to its inherently flexible metal–oxygen coordination or because it easily evolves into high-valence oxyhydroxide active phases. Conversely, an initially active amorphous material may fail rapidly if the reconstructed structure is unstable, soluble or electrically disconnected. Hence, the term “amorphous catalyst” must be used cautiously, and operando or post-operando characterization is required to verify real working structures.

### 8.2. Surface Reconstruction in Electrocatalysis: From Precatalyst to Active Oxyhydroxide

Surface reconstruction is the most typical evolution behavior of electrocatalytic materials. Under OER conditions, transition metal oxides, hydroxides, sulfides, phosphides and other materials transform into amorphous or poorly crystalline metal oxyhydroxide layers on the surface. These reconstructed layers contain high-valence metal centers, distorted M-O coordination, bound water and oxygen vacancies, which are highly favorable for OER kinetics [151,152].

The CoPi catalyst is a classic example of an in situ-formed amorphous active phase. Generated from a Co^2+^-containing phosphate electrolyte under anodic potential, it exists as a hydrated amorphous cobalt oxide/phosphate film and relies on cobalt valence variation, proton-coupled electron transfer and phosphate groups to maintain catalytic function [151,152]. XAS further confirms the existence of cobalt–oxo structural motifs in the film, proving it is an orderly local network rather than random precipitates [153].

NiFe oxyhydroxides represent another benchmark system. Fe doping dramatically boosts the OER activity of nickel-based hydroxides, and operando XAS identifies distorted octahedral Fe sites as the core active centers [114,154,155]. All evidence indicates that OER performance is governed by the working-state metal–oxygen coordination instead of long-range crystallinity.

Transition metal phosphides and sulfides are typical dual-functional water-splitting precatalysts. They exhibit excellent HER activity in the reduced state, but their surfaces oxidize into metal oxyhydroxides under anodic OER potential [156]. Taking Ni_2_P as an example, its OER activity derives from surface reconstruction rather than the original phosphide phase [157]. In this system, the precursor provides conductive cores and mechanical support, while the reconstructed amorphous shell serves as the active component.

Amorphous MoS_x_ for HER also undergoes dynamic structural evolution. Under reducing potentials, its sulfur species and Mo-S coordination continuously change along with electrochemical activation [157,158]. The real active structure is optimized gradually during operation, rather than being identical to the as-synthesized state.

### 8.3. Ion-Insertion-Induced Amorphization and Structural Reversibility in Batteries

In battery systems, structural evolution is mainly driven by ion insertion/extraction, conversion reactions, alloying/dealloying, electrolyte decomposition and mechanical stress. Controlled amorphization optimizes ion transport and strain resistance, while irreversible amorphization causes voltage hysteresis, loss of crystalline redox pathways, poor electronic conduction and severe side reactions [24,159,160].

Surface-amorphized TiO_2_ nanocrystals adopt a rational semi-amorphous structure: the disordered surface accelerates Li^+^ transport, and the crystalline core maintains structural integrity, achieving excellent high-rate lithium storage performance [161]. This proves that partial amorphization is superior to full amorphization for balancing kinetics and stability.

Amorphous vanadium oxide has outstanding sodium storage performance due to its open disordered framework, yet its flexible V-O network and bound water also bring risks of dissolution and irreversible phase transition during long cycling.

For conversion-type electrodes, cycling transforms nano-transition metal oxides into composite structures of metal nanoparticles embedded in Li_2_O matrix with high disorder [63]. Their stability depends on maintaining nanoscale contact and reversible redox at metal–oxide interfaces, instead of restoring the original crystalline phase.

Silicon-based alloying anodes fully reflect the impact of amorphization on mechanical performance. Crystalline silicon suffers from severe anisotropic expansion and fracture during lithiation, while amorphous silicon homogenizes stress distribution and suppresses crack propagation [57,88]. In situ TEM directly observes lithiation-induced structural changes in Si and SnO_2_ electrodes, verifying the strain-buffering effect of amorphization.

In aqueous zinc-ion batteries, Mn-based and V-based cathodes experience proton/Zn^2+^ insertion, dissolution–redeposition and phase transition during cycling, accompanied by the formation of amorphous phases. Local disorder and bound water facilitate Zn^2+^ migration, but uncontrolled dissolution and irreversible evolution lead to rapid capacity decay. Operando characterization technologies are necessary to distinguish reversible amorphous storage from material degradation.

### 8.4. Hydration, Coordination Change and Redox Cycling in Supercapacitors

For supercapacitors, structural evolution is limited to surface and near-surface regions during repeated redox reactions. Amorphous oxides and hydroxides rely on hydrated networks, short diffusion paths and flexible coordination to realize fast pseudocapacitance. However, long-term cycling may alter hydration degree, local bonding, pore structure and electronic connectivity.

MnO_2_-based pseudocapacitive materials store charge via proton or cation insertion and surface redox reactions, and compounding with carbon nanotubes effectively maintains electronic pathways during cycling [112]. High-rate capacitive performance requires unobstructed ion transport and accessible redox sites, which are inherent advantages of amorphous oxides.

Hydrous amorphous RuO_2_ is a classic high-performance pseudocapacitive material. Its excellent capacitance originates from fast proton–electron transfer within hydrated amorphous networks [162]. Though costly, it has guided the development of low-cost Mn, Ni, Co and Fe-based amorphous electrode materials.

For amorphous supercapacitor materials, comprehensive stability evaluation needs to cover capacitance retention, persistence of local coordination, hydrated environment and conductive networks. Operando Raman, electrochemical quartz crystal microbalance, XAS and impedance spectroscopy effectively monitor ion insertion, hydration variation and charge-transfer dynamics.

### 8.5. Amorphous Interphases in Solid-State Batteries: Protection Versus Impedance Growth

In solid-state batteries, amorphous interphases form between electrodes and solid electrolytes during operation. Beneficial interphases passivate reactive interfaces, suppress side reactions and maintain smooth ion transport; adverse interphases grow continuously, become electronically insulating, block ion migration and accumulate mechanical stress [146,163].

Amorphous LiPON forms stable interphases with lithium metal. Cryo-TEM confirms the chemically stable interphase structure at Li/LiPON interfaces, which supports the long cycle life of LiPON-based thin-film batteries [164]. Its competitiveness lies in excellent interfacial stability, rather than ultrahigh ionic conductivity.

Sulfide solid electrolytes possess high ionic conductivity and good mechanical contact with electrodes, but they are chemically reactive toward lithium metal and high-voltage cathodes. The generated interphases may either passivate interfaces or raise cell impedance continuously [165]. The stability of amorphous and glassy electrolytes must be evaluated under actual cell working conditions, including pressure, current density and cycling protocols.

Amorphous coating layers on cathode particles act as artificial protective interphases. They inhibit direct contact between active materials and electrolytes, reduce transition metal dissolution, and improve interfacial compatibility. The key design criteria are proper thickness (to guarantee ion conduction) and structural robustness (to prevent excessive interphase growth).

## 9. Challenges and Future Perspectives

Amorphous energy materials have evolved from empirical disordered phases into functional solids for electrocatalysis, batteries, supercapacitors and solid-state ionic devices. In our view, the next stage requires a change in research target: the field should no longer ask only whether a material is amorphous but which working-state disorder is useful, measurable and designable.

Future research should address five linked questions: how disorder is introduced; which local motifs and medium-range correlations remain; where amorphous regions are located; how they evolve during operation; and whether this evolution improves activity, transport and stability or accelerates degradation. These questions frame the authors’ perspective below and the specific challenges discussed thereafter.

### 9.1. Authors’ Perspective: From Amorphousness to Designable Disorder

First, the central object of future research should be operando-defined amorphous structure rather than amorphous materials in the static sense. For catalysts, this means identifying the reconstructed active layer and its formation pathway; for batteries, it means separating reversible amorphous storage from irreversible degradation; for solid-state devices, it means distinguishing ion-conducting interphases from resistive reaction layers.

Second, descriptor development should become as important as synthesis. A useful amorphous descriptor should be measurable, comparable across studies and mechanistically linked to performance. Coordination distributions, bond-length variance, medium-range connectivity, free volume, hydration state, interfacial continuity and reconstruction propensity are more valuable than a simple amorphous/crystalline label.

Third, amorphous/crystalline interfaces should be treated as design platforms rather than secondary structural features. Properly engineered interfaces can combine the flexible coordination of amorphous domains with the electronic, ionic or mechanical advantages of crystalline frameworks. The key design variables are amorphous-layer thickness, bonding strength, chemical gradient and transport resistance.

Finally, physics-informed AI should be integrated with operando characterization. Data-driven models trained only on composition and performance are unlikely to capture amorphous complexity. More useful models should include experimentally constrained descriptors and predict not only initial performance but also working-state evolution, stability and failure pathways.

### 9.2. Structural Ambiguity and Standardization of Characterization

Structural ambiguity remains the primary bottleneck. Broad XRD halos, diffuse SAED rings and absent lattice fringes are necessary but insufficient evidence because ultra-small nanocrystals, defect-rich crystals, surface-amorphized particles and beam-damaged materials may show similar signatures. Claims of amorphousness should therefore include local structure evidence and, when relevant, working-state verification.

A minimum protocol should include long-range screening by XRD/SAED/total scattering; short-range analysis by PDF, XAS, Raman and NMR; chemical and valence verification by XPS, EELS and elemental analysis; and pristine/activated/aged comparisons by operando or post-operando methods. Surface-amorphized and reconstructed materials also require depth- or interface-sensitive probes.

Reporting standards must also improve. Synthesis parameters, aging time, post-treatment, loading, film thickness, substrate, electrolyte, activation procedure and testing history should be documented because small variations can generate different amorphous states. Clear classification of genuine amorphous phases, poorly crystalline domains, defect-rich crystals, amorphous shells and operando-generated active layers is essential for reproducibility.

### 9.3. Establishment of Quantitative Structure–Performance Relationships

A second challenge is the absence of universal descriptors. Crystalline materials can be described by lattice parameters, facets and point defects, whereas amorphous materials require statistical and chemistry-aware descriptors that capture distributed local environments.

Promising descriptors include coordination-number distribution, bond-length/bond-angle variance, pair-correlation peak width, local valence distribution, M-O/M-S/M-P connectivity, free volume, hydration, medium-range order, electronic states and interfacial continuity. Electrocatalysts require adsorption energy and reconstruction descriptors, whereas batteries and ion conductors require ion-site, percolation and strain-tolerance descriptors.

Future work should integrate these descriptors with performance metrics such as overpotential, Tafel slope, Faradaic efficiency, capacity, rate capability, impedance evolution and lifetime. The comparison should shift from “amorphous versus crystalline” toward questions of disorder degree, disorder location, structural reversibility and working-state stability.

### 9.4. Amorphous/Crystalline Interface Engineering

Amorphous/crystalline interface engineering is a particularly promising route because it couples the flexible coordination and strain accommodation of amorphous phases with the conductivity, robustness and long-range transport of crystalline frameworks.

Three architectures deserve priority: crystalline-core/amorphous-shell structures for reconstructed catalysts and surface-modified battery electrodes; amorphous-domain/crystalline-backbone composites for conductive and mechanically robust electrodes; and self-limited amorphous interphases for solid-state batteries and solid oxide electrochemical devices.

The major difficulty is attribution. Enhanced performance may arise from interfacial strain, charge redistribution, altered adsorption energy, faster ion transfer, increased surface area or reconstruction chemistry. Therefore, interface studies should quantify amorphous-layer thickness, bonding strength, compositional gradients, oxygen-vacancy distribution and transport resistance using correlated microscopy, spectroscopy and impedance analysis.

### 9.5. Data-Driven and Theory-Guided Structural Modeling

Data-driven and theory-guided modeling will be indispensable because amorphous structures cannot be represented by a single periodic unit cell. Models must sample ensembles of local structures and be constrained by experimental PDF, XAS, NMR, microscopy and electrochemical data.

Molecular dynamics can simulate amorphous-network formation and ion diffusion; reverse Monte Carlo can reconstruct structures consistent with scattering data; DFT can evaluate representative local motifs; machine learning potentials can extend accessible time and length scales; and spectral learning approaches can help decode complex PDF, XAS and Raman datasets.

The most useful models will be physics-informed rather than purely correlative. They should link composition and processing to structural descriptors, and then connect those descriptors to adsorption, ion migration, interfacial resistance and degradation. Such models can provide design maps for amorphous catalysts, electrodes, solid electrolytes and SOCs.

### 9.6. Design of Operando-Defined Active Phases

The most transformative direction is the intentional design of operando-defined active phases. In many systems, the as-synthesized amorphous material is not the final functional phase but a precursor that evolves into the real active or transport structure under bias, electrolyte exposure, ion insertion or high-temperature gas gradients.

For electrocatalysis, operando Raman, XAS, FTIR, isotope tracing and online product analysis should be used to identify reconstructed oxyhydroxides, activated sulfide/phosphide motifs and true CO_2_RR/NRR active sites. For energy storage, operando PDF, XAS, Raman and impedance techniques should clarify whether amorphization is reversible, whether ion pathways remain connected and whether interphases are protective or resistive.

For solid-state batteries and solid oxide electrochemical devices, working-state analysis should track amorphous interphase growth, cation segregation, grain-boundary chemistry and electrode–electrolyte reaction layers under realistic pressure, temperature, current density and gas atmosphere. These factors determine long-term durability more directly than the pristine phase label.

The ultimate goal is controlled structural evolution: precursors should form stable active phases, amorphous interphases should be self-limiting and ion-conducting, and amorphous electrodes should preserve electronic connectivity while buffering strain. This shift from static amorphous synthesis to dynamic evolution control will define the next generation of amorphous energy materials. Table 6 systematically summarizes the major bottlenecks restricting the development of amorphous energy materials and corresponding descriptor-oriented solutions for future material design.

## 10. Conclusions

Amorphous energy materials are far more than imperfect crystalline analogs; their unique electrocatalytic and charge-storage performance stems from long-range disorder paired with ordered short/medium atomic motifs, diverse coordination environments and reversible in-operando structural evolution. Simple XRD/TEM-based “amorphous/crystalline” binary classification is misleading, as poorly crystallized, defective, surface-amorphized and electrochemically reconstructed phases differ fundamentally in structure–activity mechanisms. The core research target should be the working-state architecture defined under actual operating conditions, quantified via novel structural descriptors (coordination distribution, bond distortion, free volume, reconstruction tendency, etc.) instead of traditional crystallographic indices.

Amorphous frameworks deliver universal merits, including abundant unsaturated active sites, isotropic ion channels and strong strain buffering, widely applied in water splitting, CO_2_ reduction, batteries, supercapacitors and solid ionic devices. Yet unregulated excessive disorder causes low conductivity, material dissolution and irreversible side reactions. The optimal strategy is controllable moderate disorder rather than full amorphization. Comprehensive multimodal characterization (PDF, XAS, operando spectroscopy, and cryo-TEM) and experiment-calibrated atomistic simulations are mandatory to accurately resolve complex disordered structures, replacing single diffraction/microscopy identification.

Five core research directions are proposed: unified standards to classify various disordered phases; quantitative links between structural descriptors and electrochemical metrics; rational amorphous/crystalline interface engineering; operando tracking of structural evolution kinetics; physics-embedded data modeling to predict working-state behaviors. Solid-state batteries and solid oxide cells stand out as a vital research frontier, where amorphous interphases determine interfacial impedance and device longevity.

Overall, the field must shift from empirical amorphous synthesis to predictive structural regulation. Combined precise synthesis, multi-scale characterization, operando monitoring and quantitative modeling will convert “amorphous” from a vague structural label into a tunable design parameter, enabling energy materials with preprogrammed active sites, ion pathways and long-term operational stability.

## Figures and Tables

**Table 1:** Misconceptions and emerging consensus in amorphous energy materials.

Common Misconception	Why It Is Insufficient	Emerging Consensus/Design Implication
Amorphous = XRD halo	Broad diffraction can also arise from nanocrystals, strain, stacking disorder or beam damage.	Amorphousness requires multi-scale evidence: long-range disorder, local order and chemical identity.
The active phase is the as-synthesized phase	Many catalysts and electrodes reconstruct under bias, cycling or gas-potential gradients.	The real functional object is often the operando-defined active phase or interphase.
More disorder is always better	Excessive disorder may lower conductivity, accelerate dissolution or destabilize transport pathways.	Controlled disorder, not maximum disorder, should be optimized for each application.
Amorphous and crystalline phases should be compared as opposites	Many high-performance materials rely on amorphous/crystalline coupling.	Interfaces, shells and hybrid domains can combine active-site flexibility with electronic/ionic transport.
Descriptors from crystals can be directly transferred	Lattice parameters and facets cannot represent distributed amorphous environments.	New descriptors should include coordination distributions, bond disorder, free volume, interface continuity and reconstruction propensity.

**Table 2:** Core conceptual differences between genuine amorphous and disordered/crystalline derived materials.

Category	Long-Range Atomic Order	Key Local Structural Features	Typical Characterization Fingerprints	Electrochemical Structural Origin	Core Functional Mechanism
Genuine amorphous phases	Fully absent periodicity	Random short/medium-range coordination, uniform disordered network	Broad XRD halos, diffuse SAED rings, continuous PDF decay, featureless HRTEM	Intrinsic disorder from synthesis	Diverse unsaturated active sites, isotropic ion diffusion
Poorly crystalline materials	Partial periodicity, tiny crystallite domains	Nanoscale grain boundaries, limited coherent domains	Weak yet distinguishable Bragg peaks, discrete PDF medium-range signals	Inherent small crystal size	Exposed grain boundary active sites
Defect-rich crystalline materials	Complete lattice periodicity	Point defects, dislocations, stacking faults	Sharp XRD peaks with slight broadening, ordered PDF	Intrinsic crystal lattice defects	Defect-modulated electronic band structure
Surface-amorphized core–shell materials	Crystalline bulk core order	Disordered thin surface shell, core–shell interface	Bulk crystalline XRD signals, surface-sensitive XPS/EXAFS detects amorphous layer	Electrochemical/synthetic surface modification	Shell fast ion transport + core high conductivity
Operando-amorphized active phases	Pristine crystalline, disordered under working conditions	Dynamic reconstructed metal oxyhydroxide layers	Ex situ crystalline signals, operando PDF/Raman shows disorder	Electrochemical bias-induced reconstruction	In situ-generated flexible active coordination

**Table 3:** Five dominant performance-boosting mechanisms of amorphous energy materials.

Mechanism Category	Structural Origin	Typical Material Systems	Key Characterization Evidence	Limitation of Excessive Disorder
Abundant flexible active sites	Distorted polyhedra, dangling bonds, unsaturated metal centers	Amorphous NiFe/Co oxyhydroxides, MoS_x_	Operando XAS for variable metal coordination, Raman peak broadening	Severe material dissolution
Tunable metastable electronic structure	Distributed bond lengths, mixed valence states	RuO_2_ nanosheets, amorphous transition metal phosphides	XANES valence shift, continuous adsorption energy distribution	Reduced electronic conductivity
Isotropic ion transport pathways	Unconstrained disordered voids/free volume	Amorphous V_2_O_5_, TiO_2_, oxychloride solid electrolytes	PDF free-volume features, high ion diffusion coefficient	Irreversible ion trapping
Strain buffering capacity	Reversible local bond rearrangement	Si-based anodes, alloy-type electrode composites	In situ TEM volume evolution observation	Permanent structural collapse after long cycling
Dynamic operando reconstruction	Potential-triggered surface phase transformation	Metal sulfide/phosphide precatalysts for OER	Operando Raman/XAS tracking oxyhydroxide generation	Uncontrolled continuous degradation

**Table 4:** Main synthetic routes for amorphous energy materials.

Synthesis Strategy	Core Principle	Representative Materials	Unique Advantages	Typical Drawbacks
Wet-chemical (sol–gel/solvothermal)	Low-temperature slow condensation to suppress crystallization	Amorphous metal oxides, hydroxides, sulfides	Precise compositional tuning, scalable	Residual adsorbed water/ligands
Electrodeposition	In situ electrochemical nucleation of disordered films	CoPi, NiFeOOH, amorphous MoS_x_	Binder-free integrated electrodes	Uneven film thickness
Rapid quenching & high-energy processing	Kinetic trapping of high-energy disordered states	Metallic glasses, ball-milled amorphous electrolytes	Stable metastable disorder	Poor morphology controllability
Precursor-derived conversion (MOF/LDH/PBA)	Template inheritance followed by disorder transformation	Amorphous transition metal phosphides	Retained porous morphology	Thermal carbon impurities
In situ electrochemical amorphization	Cycling/bias-induced surface reconstruction	Crystalline sulfide/phosphide precatalysts	Active phase formed in working environment	Unpredictable reconstruction uniformity
Amorphous/crystalline hybridization	Combine disordered domains with conductive crystalline backbone	A/C-CoNiS, surface-amorphized TiO_2_ nanocrystals	Synergistic kinetics + structural stability	Complex interfacial structural identification

**Table 5:** Multimodal characterization workflow for disordered amorphous materials.

Characterization Stage	Core Techniques	Structural Information Acquired	Key Analytical Targets for Amorphous Materials
Initial long-range screening	XRD, TEM, SAED	Global periodicity, particle morphology	Distinguish broad peaks from nanocrystal strain
Local atomic structure analysis	PDF, XAS (XANES/EXAFS)	Coordination number, bond length, medium-range correlation	Quantify short/medium-range disorder degree
Local bonding & surface chemistry	Raman, FTIR, XPS, solid-state NMR	Metal–ligand vibration, element valence, ion environment	Identify M–O/M–S coordination motifs
Operando dynamic tracking	Operando Raman, operando XAS, operando PDF	Real-time structural evolution under bias	Capture reconstruction/amorphization during cycling
Auxiliary high-resolution probes	Cryo-TEM, APT, neutron scattering, DFT modeling	Interphase morphology, light element distribution	Resolve fragile beam-sensitive amorphous interphases

**Table 6:** Key research challenges and future design strategies.

Core Challenge	Detailed Description	Corresponding Descriptor-Guided Solution
Ambiguous structural identification	XRD halo signals cannot separate genuine amorphous, nanocrystals and surface-disordered materials	Multimodal joint characterization (PDF + XAS + operando spectroscopy)
Lack universal quantitative descriptors	Crystallographic lattice parameters fail for disordered systems	Adopt coordination variance, free volume, reconstruction propensity
Uncontrolled amorphous/crystalline interfaces	Random interfacial charge transfer and inconsistent kinetics	Rational core–shell/interwoven heterostructure engineering
Unpredictable operando structural evolution	Excessive disorder triggers irreversible degradation	Design controllable moderate disorder degree
Low-throughput material screening	Trial-and-error synthesis without theoretical guidance	Physics-informed machine learning structural modeling

## Data Availability

No new data were created or analyzed in this study. Data sharing is not applicable to this article.

## Abbreviations

The following abbreviations are used in this manuscript:

XRD	X-ray Diffraction
TEM	Transmission Electron Microscopy
SAED	Selected Area Electron Diffraction
PDF	Pair Distribution Function
XAS	X-ray Absorption Spectroscopy
FTIR	Fourier Transform Infrared Spectroscopy
NMR	Nuclear Magnetic Resonance
HER	Hydrogen Evolution Reaction
OER	Oxygen Evolution Reaction
ORR	Oxygen Reduction Reaction
CO_2_RR	CO_2_ Reduction Reaction
HRTEM	High-Resolution Transmission Electron Microscopy
EXAFS	Extended X-ray Absorption Fine Structure
XANES	X-ray Absorption Near-Edge Structure
M–O	Metal–Oxygen
M–S	Metal–Sulfur
XPS	X-ray Photoelectron Spectroscopy
STEM	Scanning Transmission Electron Microscopy
STEM-EELS	Scanning Transmission Electron Microscopy–Energy Loss Spectroscopy
CV	Cyclic Voltammetry
EIS	Electrochemical Impedance Spectroscopy
HEXRD	High-Energy Synchrotron X-ray Diffraction
GIPDF	Grazing-Incidence Pair Distribution Function
GIXRD	Grazing-Incidence X-ray Diffraction
APT	Atom Probe Tomography
Cryo-TEM	Cryogenic Transmission Electron Microscopy
DFT	Density Functional Theory
MOF	Metal–Organic Framework
LDH	Layered Double Hydroxide
PBA	Prussian Blue Analogue
CoPi	Cobalt Phosphate
MXene	Transition Metal Carbonitride Two-Dimensional Material
NMC	Nickel Manganese Cobalt Oxide
SEI	Solid Electrolyte Interphase
LiPON	Lithium Phosphorus Oxynitride Thin-Film Solid Electrolyte
LGPS	Li_10_GeP_2_S_12_
NRR	Nitrogen Reduction Reaction
PEC	Photoelectrochemical
EELS	Electron Energy Loss Spectroscopy
SOFC	Solid Oxide Fuel Cell
SOEC	Solid Oxide Electrolysis Cell
SOC	Solid Oxide Cell
